# Role of Volatile Organic Compounds Produced by *Kosakonia cowanii* Cp1 during Competitive Colonization Interaction against *Pectobacterium aroidearum* SM2

**DOI:** 10.3390/microorganisms12050930

**Published:** 2024-05-03

**Authors:** Mayra Paola Mena Navarro, Merle Ariadna Espinosa Bernal, Adriana Eunice Martinez-Avila, Leonela Sofia Aponte Pineda, Luis Alberto Montes Flores, Carlos Daniel Chan Ku, Yoali Fernanda Hernández Gómez, Jacqueline González Espinosa, Juan Ramiro Pacheco Aguilar, Miguel Ángel Ramos López, Jackeline Lizzeta Arvizu Gómez, Carlos Saldaña Gutierrez, José Alberto Rodríguez Morales, Aldo Amaro Reyes, José Luis Hernández Flores, Juan Campos Guillén

**Affiliations:** 1 Facultad de Química, Universidad Autónoma de Querétaro, Cerro de las Campanas s/n, Querétaro 76010, Qro, Mexico; mmena27@alumnos.uaq.mx (M.P.M.N.); mespinosa06@alumnos.uaq.mx (M.A.E.B.); adrianaeunicemartinezavila@gmail.com (A.E.M.-A.); leonelasap@gmail.com (L.S.A.P.); lmontes06@alumnos.uaq.mx (L.A.M.F.); carloschan97@outlook.es (C.D.C.K.); yhernandez01@alumnos.uaq.mx (Y.F.H.G.); jgonzalez111@alumnos.uaq.mx (J.G.E.); ramiro.pacheco@uaq.mx (J.R.P.A.); miguel.angel.ramos@uaq.mx (M.Á.R.L.); aldo.amaro@uaq.edu.mx (A.A.R.); 2 Secretaría de Investigación y Posgrado, Centro Nayarita de Innovación y Transferencia de Tecnología (CENITT), Universidad Autónoma de Nayarit, Tepic 63173, Mexico; lizzeta28@gmail.com; 3 Facultad de Ciencias Naturales, Universidad Autónoma de Querétaro, Av. De las Ciencias s/n, Querétaro 76220, Mexico; carlos.saldana@uaq.mx; 4 Facultad de Ingeniería, Universidad Autónoma de Querétaro, Cerro de las Campanas s/n, Querétaro 76010, Mexico; josealberto970@hotmail.com; 5 Centro de Investigación y de Estudios Avanzados del IPN, Irapuato 36824, Mexico

**Keywords:** biological control, volatile organic compounds, plant pathogens, virulence factors, pectinases, 2,5-dimethyl-pyrazine, acetoin, competitive colonization, *Kosakonia cowanii*, *Pectobacterium aroidearum*

## Abstract

The competitive colonization of bacteria on similar ecological niches has a significant impact during their establishment. The synthesis speeds of different chemical classes of molecules during early competitive colonization can reduce the number of competitors through metabolic effects. In this work, we demonstrate for the first time that *Kosakonia cowanii* Cp1 previously isolated from the seeds of *Capsicum pubescens* R. P. produced volatile organic compounds (VOCs) during competitive colonization against *Pectobacterium aroidearum* SM2, affecting soft rot symptoms in serrano chili (*Capsicum annuum* L.). The pathogen *P. aroidearum* SM2 was isolated from the fruits of *C. annuum* var. Serrano with soft rot symptoms. The genome of the SM2 strain carries a 5,037,920 bp chromosome with 51.46% G + C content and 4925 predicted protein-coding genes. It presents 12 genes encoding plant-cell-wall-degrading enzymes (PCDEWs), 139 genes involved in five types of secretion systems, and 16 genes related to invasion motility. Pathogenic essays showed soft rot symptoms in the fruits of *C. annuum* L., *Solanum lycopersicum*, and *Physalis philadelphica* and the tubers of *Solanum tuberosum*. During the growth phases of *K. cowanii* Cp1, a mix of VOCs was identified by means of HS-SPME-GC-MS. Of these compounds, 2,5-dimethyl-pyrazine showed bactericidal effects and synergy with acetoin during the competitive colonization of *K. cowanii* Cp1 to completely reduce soft rot symptoms. This work provides novel evidence grounding a better understanding of bacterial interactions during competitive colonization on plant tissue, where VOC synthesis is essential and has a high potential capacity to control pathogenic microorganisms in agricultural systems.

## 1. Introduction

Diverse colonization strategies are employed by the phytopathogen *Pectobacterium* spp. (Syn. *Erwinia*) to cause soft rot, slow wilt, and blackleg infections in potato plants [1,2,3,4,5,6] and bacterial soft rot in economically important crops including chili, tomato, maize, cabbage, and ornamental plants during pre-harvest or post-harvest, causing significant losses worldwide [7]. For instance, this pathogen has the ability to sense the diffusible quorum-sensing signal molecules N-acyl homoserine lactones (AHLs), which are involved in the regulation of complex pathways of positive and negative regulatory key proteins and virulence factors [8,9,10]. A principal factor affecting pathogenesis and virulence is the regulation of the production of plant-cell-wall-degrading enzymes (PCWDEs), such as pectatelyases, polygalacturonases, proteases, and cellulases, which are secreted through different secretion systems and cause severe tissue maceration [9,10]. In addition, quorum-sensing molecules regulate the production of Beta-lactam antibiotics, probably as a mechanism of bacterial competence during colonization, but their real ecological function is not known [9,11]. Molecules that are intermediate products of cell wall degradation and others not identified have also been detected as inducers of exoenzymes production [12].

Based on these colonization strategies, diverse mechanisms acting in several beneficial microorganisms have been studied for their capacity for biocontrol against *Pectobacterium* spp., and as an alternative to using chemical bactericides to control soft rot bacteria, as this has toxic effects on the environment, including alterations in communities of beneficial microorganisms [13,14,15]. One route to reduce pathogenesis and virulence is N-acyl-homoserine lactone (AHL) degradation by the *aiiA* gene that encodes a lactonase enzyme and is present in diverse microorganisms [16,17]. This strategy has been evaluated and shown significant results in reducing potato soft rot using the soil bacterium *Bacillus* that encodes a lactonase enzyme able to degrade AHLs [18]. Also, the *aiiA* gene has been cloned in plasmid pME6863 and introduced in *Pseudomonas fluorescens* P3 with similar results [19]. Genetically modified tobacco and potato plants with the *aiiA* gene also showed disease resistance against soft rot [20,21]. The use of a bacteriophage belonging to the *Podoviridae* family has also been tested as an effective mode of biocontrol for its lytic activity against *P. carotovorum* subsp. *carotovorum* [22]. On the other hand, with particular significance for our research, diverse antagonist bacterial and fungal species have been carefully assessed against *Pectobacterium* spp. [22,23]. Interestingly, cell-free filtrates obtained from diverse *Bacillus* and *Trichoderma* species have been shown to be highly effective against soft rot, which demonstrates that diverse biomolecules (known or new) can be employed to reduce infection with this plant pathogen [23].

The ability to colonize plant tissues can provide important ecological advantages during bacterial competition, but the physiological response speed can make a difference between bacterial species. Furthermore, relevant signal transduction pathways for plant–bacteria interaction are a critical factor during this period given their potential beneficial or detrimental impacts on plant tissue [24]. Therefore, it is of great significance to find beneficial bacterial species with faster colonization responses in plant tissue, with metabolic activities that have an extensive potential to control pathogenic microorganisms in agricultural systems. In this context, we recently isolated and proved the ability of *Kosakonia cowanii* to colonize *Capsicum annuum* L. tissue without causing detrimental effects [25,26]. *K. cowanii* is a member of the family *Enterobacteriaceae* with a significant metabolic ability, showing plant-promoting activities through auxin (IAA) and siderophore production [27,28], including the production of a high concentration of exopolysaccharides with effects on plant drought tolerance [28], and with the ability to produce active volatile organic compounds against certain fungal pathogens and to colonize the seeds of *C. annuum* L. [25,26]. Therefore, in the present report, we hypothesize that the plant tissue colonization competition of the *K. cowanii* Cp1 strain can reduce infection with pathogenic bacterial species. To prove this, bacterial isolates were obtained from the chili fruits of *C. annuum* L. with soft rot symptoms. One of them was identified as *P. aroidearum* SM2 by means of genomic sequencing as responsible for soft rot. Our results have shown, for the first time, the production and effects of volatile organic compounds during the colonization of *K. cowanii* Cp1 on plant tissue as a mechanism to reduce soft rot symptoms caused by *P. aroidearum* SM2. Our findings are relevant and contribute to the knowledge of the role of VOCs during colonization competition between bacteria, particularly in relation to the biocontrol of soft rot caused by *P. aroidearum*.

## 2. Materials and Methods

### 2.1. Isolation of Pathogen Organism

The infected fruits of *Capsicum annuum* var. Serrano with soft rot symptoms were obtained from a local market at Querétaro, México. The damaged tissue was scraped with an inoculation loop under sterile conditions and spread on the surface of tryptic soy agar (TSA) medium (Difco Laboratories; Detroit, MI, USA) and incubated for 24 h at 37 °C. The bacterial colonies obtained were tested according to Koch’s postulates on healthy serrano chili fruits obtained from a local market in Querétaro, México. The healthy chili fruits were surface-sterilized via immersion in 10% (*v*/*v*) sodium hypochlorite for 10 min, then in 75% (*v*/*v*) ethanol for 10 min, and finally dried under sterile conditions. Each bacterial isolate was prepared in 0.1% peptone at a dilution of 1 × 10^8^ UFC/mL [29] and vortexed for 1 min, then 10 µL of each bacterial suspension was inoculated in triplicate on the chili fruits’ surfaces, which were punctured with a sterile needle and inoculated at that point. Negative controls were inoculated with 0.1% peptone. The inoculated chili fruits were placed inside an airtight container at room temperature (25 °C) and natural sunlight conditions. The experiment was performed in duplicate, and bacterial colonies that did not present infection symptoms during the 3 days that they were observed in chili fruits were discarded.

### 2.2. Pathogenicity Test

According to the pathogenicity test, the bacterial colony of the SM2 strain had the ability to cause soft rot in serrano chili fruits. As such, additional pathogenicity tests were conducted in healthy fruits of Jalapeño pepper (*Capsicum annuum*), tomato (*Solanum lycopersicum*), serrano chili (*Capsicum annum*), green tomato (*Physalis philadelphica*), and potato tubers (*Solanum tuberosum*) following the same methodology as above. The fruits and tubers inoculated were assessed every 12 h for 48 h. The calculation of the minimum inoculum required to cause infection was carried out by inoculating 10 µL of different dilutions of SM2 strain from 1 × 10^−1^ to 1 × 10^−8^ UFC/mL in tomato and chili fruits. They were observed for 48 h to determine the minimum inoculum dilution able to cause soft rot symptoms [30].

For the pathogenicity test on *Capsicum annuum* ver. Poblano plants, a suspension of SM2 strain was prepared using a concentration of 1 × 10^8^ CFU/mL in 0.1% peptone. Serrano chili plants of 3 months of age were infected with the SM2 strain. The leaves of the seedlings were pricked with a sterile hypodermic needle, and in each wound, 10 µL of bacterial suspension was placed. For each treatment, including the controls (0.1% peptone), three leaves of the seedling were infected, and this was performed in duplicate. Inoculated seedlings were grown in greenhouse conditions (24 °C, 50% relative humidity and natural sunlight) and observed for 30 days [30].

### 2.3. Identification of Bacterial Isolate SM2

For bacterial identification, the genomic DNA of the SM2 strain was isolated using the ZymoBIOMICS^TM^ DNA Miniprep Kit (Zymo Research, Irvine, CA, USA) following the manufacturer’s protocol. Subsequently, genomic sequencing was performed at Zymo Research, Irvine, CA, USA, utilizing the NovaSeq^®^ platform (Illumina, San Diego, CA, USA). The Sequence Read Archive data of the SM2 strain were submitted to the Comprehensive Genome Analysis Service, using PATRIC at the Bacterial and Viral Bioinformatics Resource Center (BV-BRC); for data filtration, TrimGalore, CutAdapt, Fastq-Pair, and FastQC were used [31], SPAdes was used for assembly, and the RAST tool kit (RASTtk) was used for genome annotation [31,32]. Proksee Assemble, CGView Builder, CARD RGI, and Prokka [33] were utilized to construct a circular genome map. To generate bacterial phylogenetic trees, the Codon Tree pipeline at BV-BRC was utilized with Mash/MinHash [34], and global protein families (PGFams) were analyzed with MUSCLE v5 [35] and processed with RAxML v8.2.11 (Randomized Axelerated Maximum Likelihood) [36] to determine the phylogenetic analysis. JSpeciesWS [37] was used to measure the probability of whether the genome belonged to the same species by a pairwise comparison of their Average Nucleotide Identity (ANI). DNA sequences were deposited in NCBI with BioSample accession: SAMN40918343. Accession number JBCFOM000000000.

### 2.4. Antibiotics Sensitivity Test

The test of antibiotic susceptibility to SM2 was conducted following the disk diffusion method outlined in the CLSI guidelines (Clinical and Laboratory Standards Institute: CLSI Guidelines) [38]. Eighteen commercially available antibiotic discs (Thermo Scientific^TM^-Oxoid^TM^, Santa Fe, NM, USA) were applied to the Mueller–Hinton agar (BD Bioxon, Ciudad de Mexico, Mexico) surface, including clindamycin (30 µg), dicloxacillin (1 µg), erythromycin (15 µg), penicillin (10 U), tetracycline (30 µg), vancomycin (30 µg), amikacin (30 µg), ampicillin (10 µg), carbenicillin (100 µg), cephalothin (30 µg), cefotaxime (30 µg), ciprofloxacin (5 µg), chloramphenicol (30 µg), gentamicin (10 µg), netilmicin (30 µg), nitrofurantoin (300 µg), norfloxacin (10 µg), and sulfamethoxazole/trimethoprim (25 µg). After incubation at 37 °C for 18–24 h, the diameters of the inhibition zones surrounding each antibiotic disc were measured in millimeters by duplicate.

### 2.5. Bacterial Competitive Colonization Interaction

Bacterial competitive colonization interaction assays were performed on healthy fruits of tomato (*Solanum lycopersicum*) and jalapeño chili (*Capsicum annum*) to assess the potential of the strain *K. cowanii* Cp1 (previously isolated and characterized [26]) against the *P. aroidearum* SM2 strain and its ability to reduce soft rot symptoms. The fruits were inoculated as previously described above in the infection methodology, with 10 µL of each bacterial suspension added at a concentration of 1 × 10^8^ CFU/mL, according to the treatments shown in Table 1. The interactions of *K. cowanii* Cp1 and *P. aroidearum* SM2, inoculated at the same time (T1), were evaluated. Similarly, *P. aroidearum* SM2 was inoculated first (time 0), followed by *K. cowanii* Cp1 at 3 and 6 h (T2 and T3), to evaluate pathogen establishment; in turn, *K. cowanii* Cp1 was inoculated first (time 0), followed by *P. aroidearum* SM2 at 3, 6, and 9 h (T4, T5, and T6). The controls were inoculated only with *K. cowanii* Cp1 (T7), *P. aroidearum* SM2 (T8), and 0.1% peptone (T9). The inoculated fruits were placed inside an airtight container at room temperature (25 °C) and natural sunlight conditions. The treatments were observed for 48 h to determine the soft rot symptoms.

### 2.6. Bacterial Growth Inhibition Essays on Agar Plates

To determine whether *K. cowanni* Cp1 produces biomolecules with the capacity to inhibit the growth of the pathogenic *P. aroidearum* SM2, different assays on agar plates were performed. First, two drops of 10 µL (1 × 10^8^ CFU/mL) of both microorganisms were placed on TSA medium (Difco Laboratories; Detroit, MI, USA) separated by 0.5 cm to evaluate the inhibition zone around the bacterial colony. Secondly, we carried out the evaluation of a cell-free filtrate obtained via centrifugation and filtration with 0.22 µm pore size filters (Corning Incorporated) from a culture of *K. cowanni* Cp1 during 10 h of growth on tryptic soy broth medium (Difco Laboratories; Detroit, MI, USA). The test of the cell-free filtrate’s susceptibility towards *P. aroidearum* SM2 was conducted via the disk diffusion method, where paper filters were impregnated with 10 µL of cell-free filtrate at 2, 4, 6, 8, and 10 h of bacterial growth at 37 °C and 120 rpm and placed on TSA medium (Difco Laboratories; Detroit, MI, USA) inoculated with 100 µL (1 × 10^6^ CFU/mL) of *P. aroidearum* SM2 and incubated at 37 °C for 24 h [39,40]. Third, to determine whether volatile organic compounds (VOCs) could inhibit the growth on the pathogenic *P. aroidearum* SM2, the double culture method described previously was used [26], as illustrated in the Supplementary Materials (Figure S1). In this experiment, a two-compartment Petri dish plate device was used; on the bottom side of this device, *K*. *cowanii* Cp1 was inoculated at 100 μL (10^8^ CFU/mL) onto TSA medium (Difco Laboratories; Detroit, MI, USA) and grown for 2, 4, or 6 h at 37 °C to allow time to produce any VOCs. After this period of time, *P. aroidearum* SM2 was inoculated onto the TSA medium (Difco Laboratories; Detroit, MI, USA) at 10 µL serial dilutions (10^−1^ to 10^−6^) on the bottom side of the compartment so as to evaluate colony growth inhibition. For the negative control, only the *P. aroidearum* SM2 suspension was inoculated on the TSA agar. The two-compartment plastic plate device was sealed with parafilm and incubated at 37 °C for 24 h. All assays were carried out in duplicate. The diameters of the *P. aroidearum* SM2 colonies were measured to observe the inhibition of their growth by *K. cowanii* Cp1 VOCs. The data were analyzed using Tukey’s mean adjustment test (*p* = 0.05), which was performed using the Minitab Statistical Software Version 21.1.0.

### 2.7. Pathogenic Inhibition Assays Using Cell-Free Filtrate on Chili Fruits

We aimed to determine whether a cell-free filtrate of *K. cowanii* Cp1 contained biomolecules with the ability to reduce the infection of *P. aroidearum* SM2 on serrano chili fruits. To test this, 1 mL samples of cell-free filtrate of bacteria grown for 0, 1, 2, 3, 4, 6, 8, and 10 h on tryptic soy broth medium (Difco Laboratories; Detroit, MI, USA) at 37 °C and 120 rpm were placed into 50 mL Falcon tubes. These cell-free filtrates were inoculated with 10 µL of a bacterial suspension (10^8^ CFU/mL) of *P. aroidearum* SM2. In each treatment, a disinfected serrano chili fruit with a tissue wound was introduced into the Falcon tube to be infected with *P. aroidearum* SM2. Additionally, both a negative (0.1% peptone) and a positive control (*P. aroidearum* SM2) inoculated in 0.1% peptone were included. The development of soft rot was evaluated for 48 h, as illustrated in the Supplementary Materials (Figure S2).

### 2.8. Identification of VOCs Using HS-SPME-GC-MS

To test the hypothesis that the initial competitive colonization of *K. cowanii* Cp1 on plant tissue produces a diffusible volatile organic compound with the ability to reduce infection with *P. aroidearum* SM2, the profiles of VOCs were characterized during bacterial growth phases. First, a bacterial growth curve was constructed to determine the times of the bacterial growth phases. Second, based on previous experiments’ results and growth curves, we decided to perform VOC analysis during the lag phase (2 h), log phase (4 h), and stationary phase (6 h). To do this, 40 mL of tryptic soy broth medium (Difco Laboratories; Detroit, MI, USA) was inoculated with the *K. cowanii* Cp1 strain (10^8^ CFU/mL) in borosilicate glass media bottles of 80 mL (Schott) and incubated for 2, 4, and 6 h at 37 °C and 120 rpm. The tryptic soy medium without the bacterial strain was used as a control. Once the bacterial cultures were obtained, the VOCs were analyzed using the following methodology [26]: the samples were incubated at 50 °C for one hour, and then the VOCs were collected on a divinylbenzene/carboxen/polydimethylsiloxane fiber (DVB/CAR/PDMS, Supelco, Sigma-Aldrich, Visalia, CA, USA). After this, manual injection was carried out in splitless mode; the injection port and transfer line temperature were set to 250 °C using a 7820A GC with a 5975C MSD (Agilent Technologies, Inc., Santa Clara, CA, USA) and HP-5MS 30 m, 0.25 mm, and 0.25 µm GC Column Capillaries (Agilent Technologies Inc., Santa Clara, CA, USA). The column oven was programmed at 40 °C, increasing to 180 °C at 5 °C/min, and then at 20 °C/min to 260 °C and held at that temperature for 5 min. Helium (99.999% purity) was used as the carrier gas with a flow rate of 1.0 mL/min. Mass spectrometry analyses were conducted at an electron energy of 70 eV, and the *m*/*z* range was 33–500. Data were obtained and processed using NIST/EPA/NIH Mass Spectra Library instrumental analysis software, version 2017, Antioch, CA, USA.

### 2.9. Evaluation of Pathogenic Inhibition by Standard VOCs

After the VOCs’ characterization, we evaluated commercially available 2,5-dimethylpyrazine (98%, Sigma-Aldrich, USA) and acetoin (≥95%, Sigma-Aldrich, USA), which showed high relative peak-area percentages during bacterial growth phases. First, to test their bactericidal effects on both bacterial strains, the volatile organic compounds were evaluated on TSA plates inoculated with each bacterial species, with different volume concentrations (5, 10, and 20 µL) applied on sterile filter paper discs to measure the area of inhibition. Subsequently, these compounds were tested on disinfected chili peppers following the methodology outlined previously. Three wounds were made per chili fruit using a sterilized needle. The treatments were T1, represented by the negative control with a bacterial suspension of *K. cowanii* Cp1 (10 µL); T2, which served as the positive control with the inoculation of 10 µL of *P. aroidearum* SM2. Both bacterial suspensions were of 10^8^ CFU/mL in 80 µL of 0.1% peptone. For T3, a suspension was prepared with 80 µL of 0.1% peptone, 10 µL of *K. cowanii* Cp1 bacterial suspension (10^8^ CFU/mL), and 10 µL of *P. aroidearum* SM2 bacterial suspension (10^8^ CFU/mL); from this bacterial mix, 10 µL of sample was inoculated. For each treatment from T4 to T10, a suspension containing 80 µL of 0.1% peptone, 10 µL of *K. cowanii* Cp1 bacterial suspension (10^8^ CFU/mL), and 10 µL of *P. aroidearum* SM2 bacterial suspension was prepared (10^8^ CFU/mL). In these bacterial suspensions, the volume concentrations of the compounds were added as follows: T4 to T6 received 5, 10, and 20 µL of 2,5-dimethyl-pyrazine, respectively; T7 to T9 received 5, 10, and 20 µL of acetoin, respectively. T10 received 5 µL of acetoin and 5 µL of 2,5-dimethyl-pyrazine. For T11, a solution was prepared with 80 µL of 0.1% peptone, 10 µL of *P. aroidearum* SM2 bacterial suspension (10^8^ CFU/mL), and 20 µL of 2,5-dimethyl-pyrazine, while for T12, the solution contained 80 µL of 0.1% peptone, 10 µL of *P. aroidearum* SM2 bacterial suspension (10^8^ CFU/mL), and 20 µL of acetoin. Finally, for T13 and T14, the controls, 20 µL of 2,5-dimethyl-pyrazine and 20 µL of acetoin, respectively, were added. In all these treatments, 10 µL of sample was inoculated.

## 3. Results

### 3.1. Isolation of Pathogen Organism

**Figure 1:**
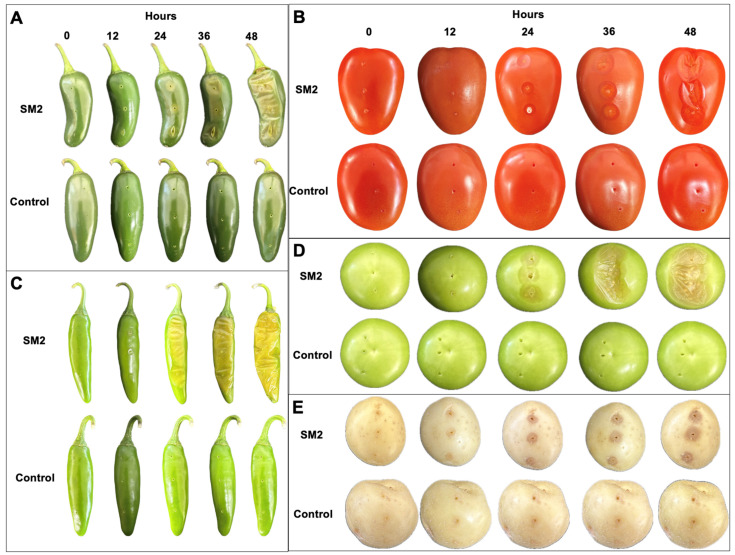
Evaluation of the infective capacity of SM2 isolate. Fruits and tubers were inoculated with bacterial suspension at a concentration of 10^8^ CFU/mL. (**A**) Jalapeño pepper (*Capsicum annum*); (**B**) tomato (*Solanum lycopersicum*); (**C**) serrano chili (*Capsicum annuum*); (**D**) green tomato (*Physalis ixocarpa*); (**E**) potato (*Solanum tuberosum*). Over 48 h, the soft rot symptoms were registered.

The bacterial isolation was conducted to obtain pathogenic species causing soft rot in the fruits of serrano chili. Five bacterial colonies with different phenotypes were obtained on TSA medium, and following Koch’s postulates, only the SM2 isolate showed soft rot symptoms and was selected for further characterization. The pathogenicity test results show that the SM2 isolate had severe effects, causing soft rot in all the fruits and tubers used. The results are shown in Figure 1; in all cases, the SM2 isolate showed an infective capacity and gave rise to clear soft rot symptoms 24 h post-inoculation, with complete infection observed at 48 h in chili (Figure 1A,C), tomato (Figure 1B), and green tomato (Figure 1D) fruits compared with the controls. In potato tubers, the soft rot symptoms were moderate during the 48 h of observation (Figure 1E) compared with the control. Interestingly, when pathogenic tests were performed on the leaves of *Capsicum annuum* var. Poblano plants, no visible evidence of infection was observed, at least for this specific cultivar (Supplementary results, Figure S3). When the infection on the fruits and tubers was tested, we aimed to determine the minimum quantity of inoculum that would cause soft rot. Therefore, bacterial dilutions were tested (1 × 10^−1^ to 1 × 10^−8^), and 10 µL of bacterial suspension was inoculated onto serrano chili (*Capsicum annuum* var. Serrano), jalapeño chili (*Capsicum annuum* var. Jalapeño), and tomato (*Solanum lycopersicum*). After 48 h, soft rot symptoms were observed up to the dilution 1 × 10^−6^ (Supplementary results Figure S4), with approximately 100 CFU in 10 µL of bacterial suspension inoculated. All these results show that the SM2 isolate may have important implications during the pre-harvest or post-harvest periods of economically important crops. Therefore, we decided to identify SM2 through genome sequencing.

### 3.2. Genomic Characterization of SM2 Isolate

**Figure 2:**
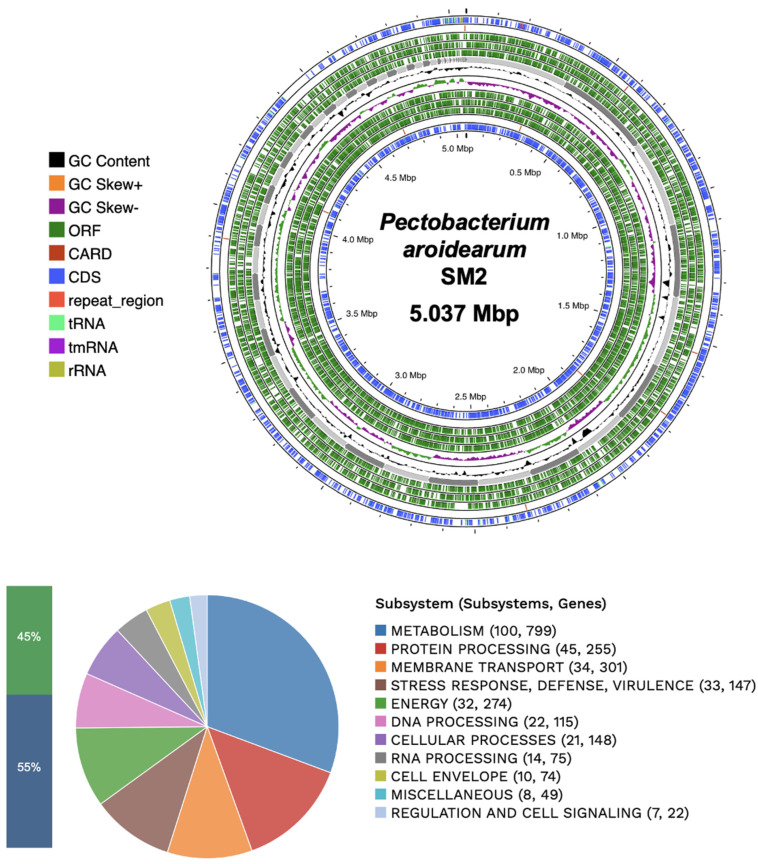
Circular genomic map and subsystems information for *P. aroidearum* SM2. From the outside to the center are the ensemble contigs, with CDSs in the front strand, ORF on the reverse strand, RNA genes, CDSs with similarity to known antibiotic resistance genes, CDSs with similarity to virulence factors, GC content, and GC skew. The distributions of the subsystems are displayed in the figure below. In terms of subsystems coverage, 45% indicates a total of 2269 genes and 55% represents those not indicated in the subsystem average, with a total of 2829 genes.

**Figure 3:**
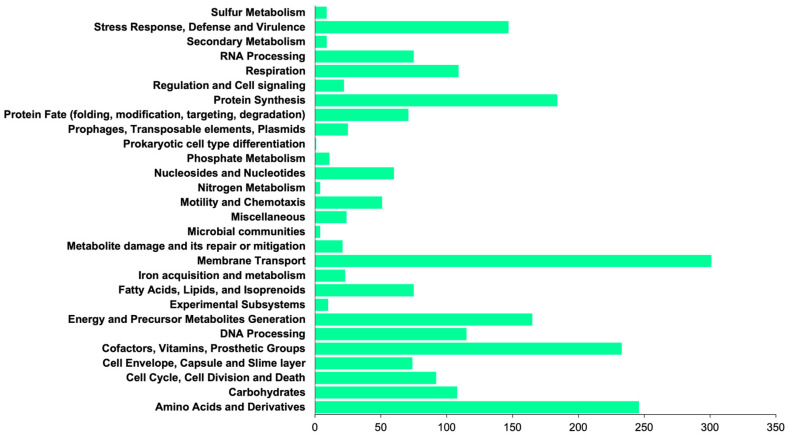
Count of subsystems by category. Annotation of the various primordial metabolic processes of the *P. aroidearum* strain SM2.

The sequence read data of the SM2 isolate were submitted to the PATRIC Comprehensive Genome Analysis Service. According to the complete analysis, the SM2 isolate was identified as *Pectobacterium aroidearum* SM2. The genome quality assessment showed coarse consistency (98.5), fine consistency (97.6), CheckM completeness (100), and CheckM contamination (0.3), suggesting that this genome is of good quality. The genome contains 86 contigs with an estimated length of 5,037,920 bp and an average guanine-cytosine content of 51.46%. According to the data provided by RASTtk, the genome has 4925 protein coding sequences (CDSs), 69 transfer RNA (rRNA) genes, and four ribosomal RNA (rRNA) genes. Additionally, the genome of *P. aroidearum* SM2 shows similarities to other known genes (Table 2), such as the virulence factors (Victors, VFDB, PATRIC), antibiotic resistance genes (PATRIC, CARD), transporters (TCDB), and drug target genes (DrugBank) that were identified in different databases. In addition, the genome annotation of the proteins with subsystem functional assignments is shown in Figure 2. The distribution of the subsystems by the category of *P. aroidearum* SM2 is shown in Figure 3.

**Figure 4:**
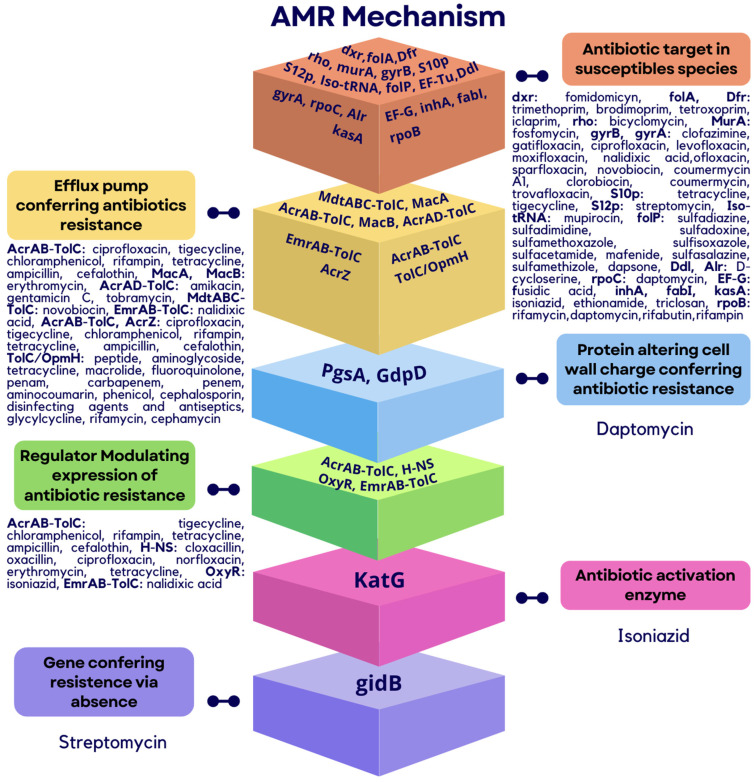
Antibiotic resistance genes. The AMR mechanism and its related genes are indicated with color diagrams.

According to the analysis of the genome of *P. aroidearum* SM2, the predicted antibiotic resistance genes were grouped into six different mechanisms of action, as depicted in Figure 4. Antibiotic targets in susceptible species were represented by the following genes: *dxr*, *folA*, *dfr*, *rho*, *murA*, *gyrB*, *s10p*, *s12p*, *iso-tRNA*, *folP*, *eF-tu*, *ddl*, *gyrA*, *rpoC*, *alr*, *eF-G*, *inhA*, *fabl*, *kasA*, and *rpoB*. Regarding the mechanism of efflux pump conferring antibiotics resistance, the genes detected were *mdtABC-TolC*, *macA*, *acrAB-TolC*, *macB*, *acrAD-TolC*, *emrAB-TolC*, *acrAB-TolC*, *acrZ,* and *tolC/opmH*. Regarding proteins altering the cell wall charge and conferring antibiotic resistance, only two genes were found: *psgA* and *gdpD*. Regulators modulating the expression of antibiotic resistance were represented by *acrAB-TolC*, *h-NS*, *oxyR*, and *emrAB-TolC*. The antibiotic activation enzyme and the gene conferring resistance via absence were represented by a single gene each: *katG* and *gidB*, respectively.

However, when the AMR phenotype was tested, the results showed resistance only to erythromycin, while sensitivity was observed for ampicillin, cephalothin, cefotaxime, ciprofloxacin, clindamycin, dicloxacillin, gentamicin, penicillin, tetracycline, sulfamethoxazole/trimethoprim, vancomycin, amikacin, carbenicillin, chloramphenicol, netilmicin, and nitrofurantoin. These results demonstrate the importance of antibiotic resistance phenotype assays versus genotype annotation for genome analysis.

The virulence genes were predicted using Victors, PATRIC, and VFDB sources, which are important databases for predicting pathogenicity. In the Victors database, a total of 81 virulence genes were detected; from the PATRIC database, we obtained a total of 49 virulence genes, and in the VFDB database, we detected a total of 17 virulence genes. Furthermore, we searched for relevant genes encoding plant-cell-wall-degrading enzymes (PCDEWs), toxins, and secretion systems for *P. aroidearum* SM2 (Table 3). We found 12 genes that encode PCDEWs. Also, we discovered 139 genes involved in five types of secretion systems: 22 genes in type I (T1SS), 22 genes in type II (T2SS), 40 genes in type III (T3SS), 19 genes in type IV (T4SS), none in type V (T5SS), and 20 genes in type VI (T6SS). On the other hand, 16 genes were related to invasion motility.

**Figure 5:**
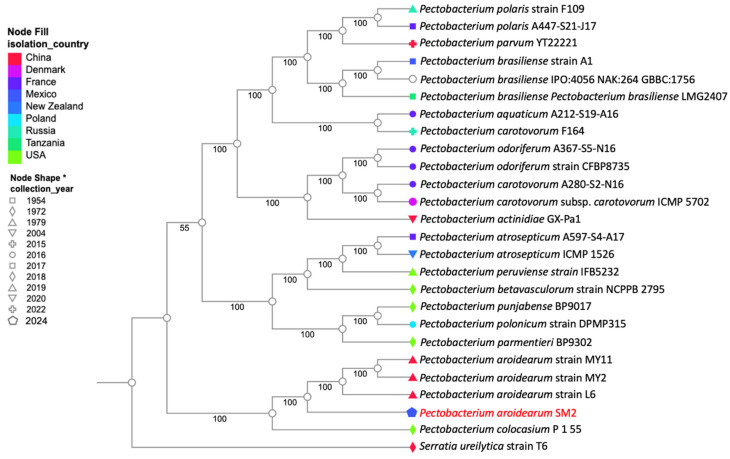
Phylogenomic analysis of *P. aroidearum* SM2. Phylogenetic analysis for species of *Pectobacterium* genus was performed in BV-BRC. The node shape represents the collection year. The node color represents the country from which the isolates were collected, including Mexico, China, Denmark, France, Poland, Russia, Tanzania, the USA, and New Zealand. *Serratia ureilytica* strain T6 was included as an outgroup.

According to the phylogenetic tree (Figure 5), *P. aroidearum* SM2 matches with the clade that includes *P. aroidearum* strain MY2, which was isolated from *Amorphophallus konjac* in China, as well as with *P. aroidearum* L6 isolated from the soft rot of *S. podophyllum* in Hainan province, China. It also matches with the *P. aroidearum* strain MY11 isolated from a tuber (*Amorphophallus konjac*) from Sichuan province, China. The ANI values confirmed these phylogenetic analyses (Supplementary results, Table S1). The pairwise genome comparison of *P. aroidearum* SM2 showed ANI values of 97.30% with *P. aroidearum* L6 and 97.25% with *P. aroidearum* QJ002. Therefore, the genome of *P. aroidearum* SM2 is of great relevance to understanding soft rot in the fruits of chili and tomato, as well as in potato tubers, in the Mexican context, as these are crops of economic importance.

### 3.3. Bacterial Competitive Colonization Interaction

**Figure 6:**
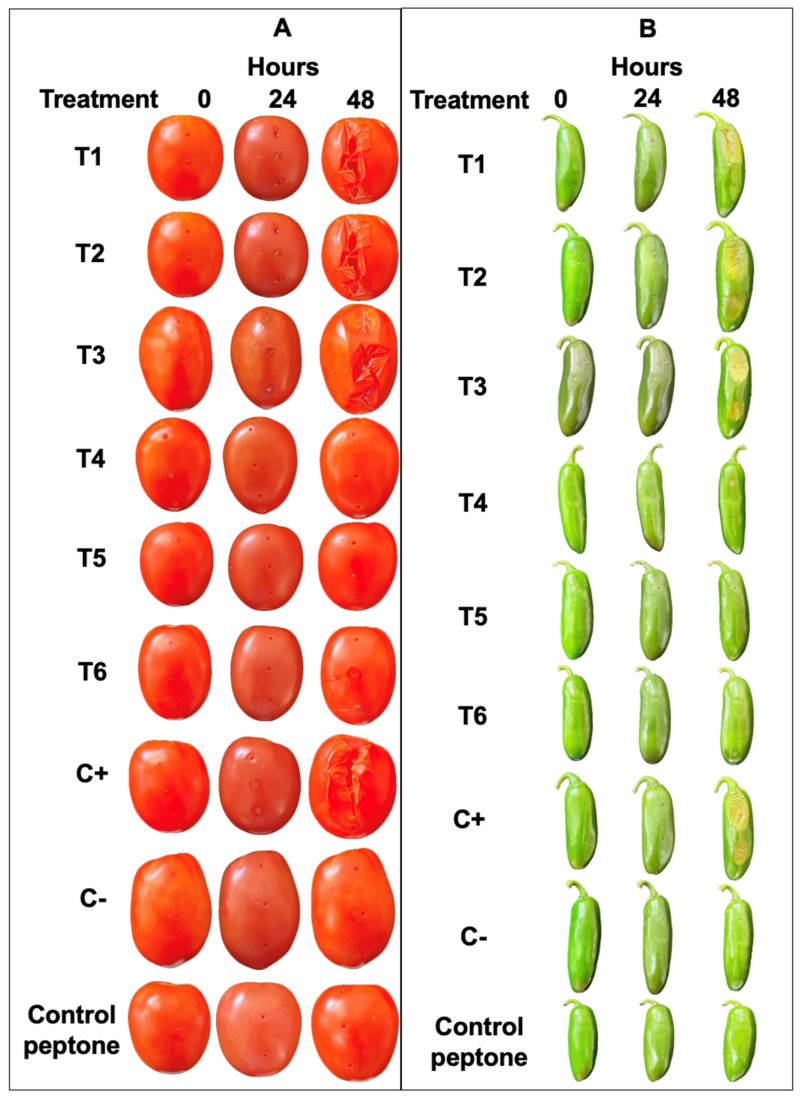
Competitive colonization interaction between *K. cowanii* Cp1 and *P. aroidearum* SM2 on tomato (*Solanum lycopersicum*) (**A**) and jalapeño chili (*Capsicum annum*) (**B**). See methodology for the treatments.

Competitive colonization interaction assays were carried out between *K. cowanii* Cp1 and *P. aroidearum* SM2 to evaluate their ability to reduce soft rot symptoms in the fruits of tomato and jalapeño chili (Figure 6). The results show that when both bacterial species were inoculated at the same time (T1) or post-inoculated with *K. cowanii* Cp1 at 3 (T2) and 6 h (T3), soft rot symptoms were evident at 24 h and severe at 48 h in the fruits of tomato and jalapeño chili. These results are similar to those for the positive control inoculated with *P. aroidearum* SM2, while for *K. cowanii* Cp1 (negative control) and the control with 0.1% peptone, soft rot symptoms were not observed. This result indicates that *P. aroidearum* SM2 probably achieves faster colonization, causing severe tissue maceration, and the presence of *K. cowanii* Cp1 has no effect on competitive colonization. Therefore, we evaluated whether *K. cowanii* Cp1 requires more time to achieve competitive colonization. Interestingly, our findings show that when *K. cowanii* Cp1 was inoculated first and *P. aroidearum* SM2 was applied after 3 (T4), 6 (T5), or 9 (T6) hours, soft rot symptoms were not observed during the evaluation period in the fruits of tomato and jalapeño chili. Therefore, our results show that *K. cowanii* Cp1 probably requires time to achieve competitive colonization through the synthesis of a biomolecule with a fast diffusion ability in plant tissue, and with an inhibitory ability to reduce soft rot symptoms caused by *P. aroidearum* SM2. To prove this hypothesis, we developed different microbiological assays to identify these biomolecules.

### 3.4. Bacterial Growth Inhibition Assays on Agar Plates

**Figure 7:**
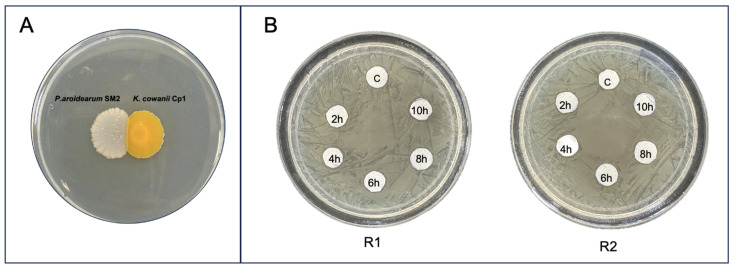
Evaluation of bacterial growth inhibition essays. (**A**) *K. cowanii* Cp1 and *P. aroidearum* SM2 in confrontation and (**B**) cell-free filtrate tests performed at 2, 4, 6, 8, and 10 h of growth of *K. cowanii* Cp1, evaluated directly on *P. aroidearum* SM2 in duplicate (R1 and R2).

**Figure 8:**
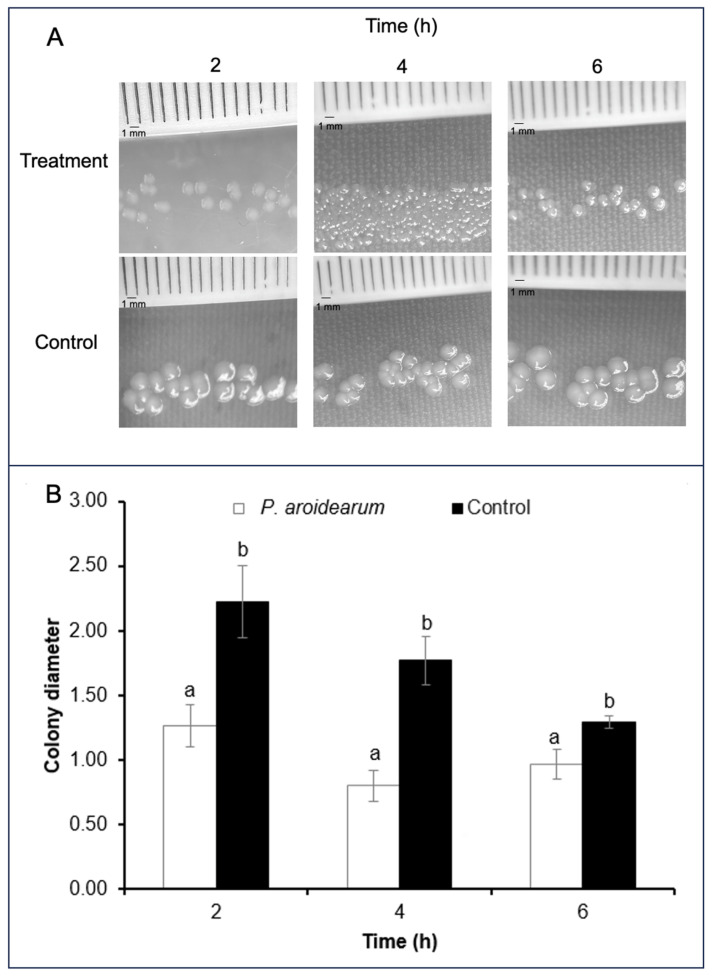
Inhibition of bacterial growth colonies of *P. aroidearum* SM2 through VOCs produced by *K. cowanii* Cp1. (**A**) Bacterial growth colonies produced using the double culture method and (**B**) measurement of colony diameter at 2, 4, and 6 h of experimental growth. The data were analyzed using Tukey’s mean adjustment test (*p* = 0.05), which was performed in the Minitab Statistical Software. Values by different letters show statistical differences.

To obtain information about a biomolecule with the potential ability to inhibit growth on *P. aroidearum* SM2, different bacterial growth assays were constructed. First, in vitro confrontations between both bacterial species were set up on TSA medium (Figure 7A) without any evident growth-inhibitory result. After that, cell-free filtrates were obtained from a culture of *K. cowanni* Cp1 that permitted 10 h of growth on tryptic soy broth medium. However, these cell-free filtrates showed no growth inhibitory effects on *P. aroidearum* SM2 cultivated on TSA medium (Figure 7B). These results suggest null growth inhibition under our experimental conditions, and probably lower concentrations of a biomolecule with potential growth inhibition capacity. Moreover, we decided to use the double culture method and grow *K. cowanni* Cp1 for different times on TSA medium (bottom side of the compartment), permitting 2, 4, and 6 h to produce volatile organic compounds. After these periods of time, *P. aroidearum* SM2 was inoculated at serial dilutions on TSA medium (upper side of the compartment) and incubated at 37 °C for 24 h to obtain colonies, after which we evaluated the impacts on colony growth. The results in Figure 8A show that the VOCs produced by *K. cowanii* Cp1 in the TSA medium affected the size of the bacterial growth colonies of *P. aroidearum* SM2 compared with the control for all the time periods evaluated. The diameters of *P. aroidearum* SM2 colonies inoculated at 2, 4, and 6 h on *K. cowanii* Cp1 were significantly smaller compared to the diameters of *P. aroidearum* SM2 grown on control plates (Figure 8B). Thus, a greater reduction in colony sizes was observed at 2 and 4 h of inoculation, whereas the difference in the diameters of *P. aroidearum* SM2 compared to the control was ~50%. At 6 h, the difference between the diameters was ~30%. This interesting finding proves that cell-free filtrates can be used directly in vivo to evaluate soft rot reduction in serrano chili.

### 3.5. Pathogenic Inhibition Assays Using Cell-Free Filtrate on Chili Fruits

**Figure 9:**
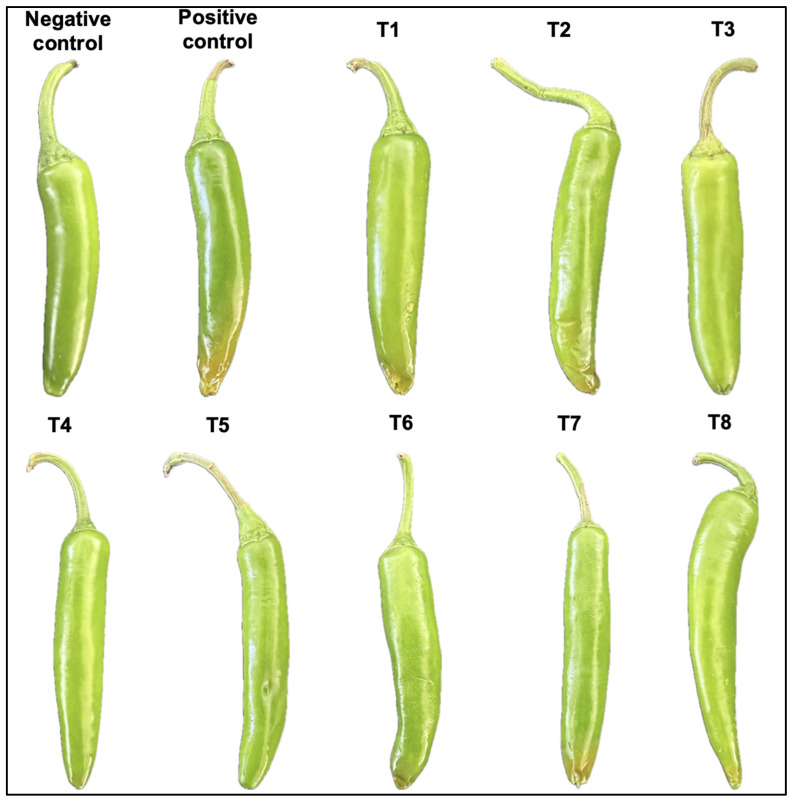
Pathogenic inhibition assays using cell-free filtrates obtained from bacterial growth of *K. cowanii* Cp1. Cell-free filtrates were obtained at 0 (T1), 1 (T2), 2 (T3), 3 (T4), 4 (T5), 6 (T6), 8 (T7), and 10 h (T8). *P. aroidearum* SM2 was inoculated in 1 mL of each cell-free filtrate, and serrano chili fruit was immersed directly using a 50 mL Falcon tube in order to evaluate the reduction in soft rot symptoms over 24 h. For negative and positive controls, we used 1 mL of 0.1% peptone. Experiments were performed in triplicate.

Based on the previous results and to increase the concentration of any potential biomolecule produced by *K. cowanii* Cp1, we decided to use 1 mL of cell-free filtrate obtained via 10 h of bacterial growth on tryptic soy broth medium and placed this into 50 mL Falcon tubes, in which we directly immersed serrano chili to evaluate the reduction in soft rot symptoms caused by *P. aroidearum* SM2. The results shown in Figure 9 are remarkable; cell-free filtrates obtained at 0 (T1) and 1 h (T2) of bacterial growth showed soft rot symptoms compared with the positive control, while cell-free filtrates obtained at 2 (T3), 3 (T4), and 4 h (T5) showed inhibited soft rot symptoms compared with the positive and negative controls. On the other hand, cell-free filtrates obtained at 6 (T6), 8 (T7), and 10 h (T8) also showed soft rot symptoms. These results demonstrate that *K. cowanii* Cp1 probably produces volatile organic compounds during competitive colonization to increase its capacity to evade competitors, but the synthesis of these biomolecules requires a certain period of time after colonization. Therefore, we undertook the characterization of VOCs during bacterial growth phases to identify these molecules.

### 3.6. Identification of VOCs Using HS-SPME-GC-MS

**Figure 10:**
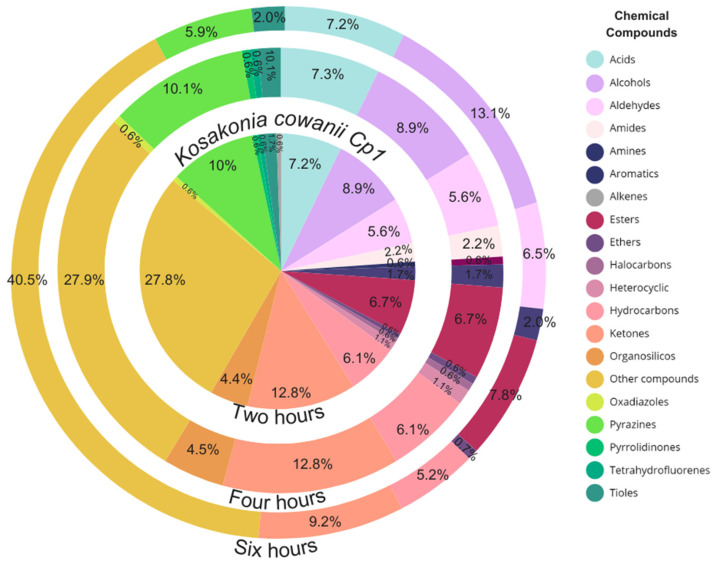
Characterization by means of HS-SPME-GC-MS of VOCs produced by *K. cowanii* Cp1 during bacterial growth phases. The colors in the graph represent the chemical classes of the VOCs identified as relative peak-area percentages.

**Figure 11:**
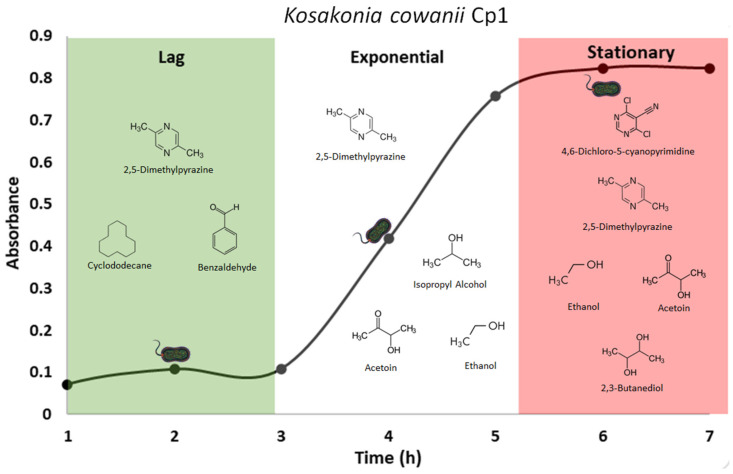
VOCs with highest relative peak-area percentages produced by *K. cowanii* Cp1 during growth phases. The bacterial cells represent the time of sampling (2, 4, and 6 h) during growth for VOC analysis using HS-SPME-GC-MS.

Based on the bacterial growth curve registered on tryptic soy broth medium and the reduction in soft rot symptoms revealed by *K. cowanii* Cp1, we used the characterization of VOCs during the lag phase as an approximation of which biomolecules are present during the early stages of competitive colonization, and those during log-stationary phases were used to assess the differences in VOCs between physiological growth phases. Together, this reveals the true nature of these important metabolic activities that are enacted during colony establishment. The results obtained by means of HS-SPME-GC-MS were highly significant (Figure 10 and Supplementary Tables S2–S4 and Figure S5. The VOCs characterized at 2 h of bacterial growth (lag phase) included chemical classes of ketones (10.38%), esters (9.84%), pyrazines (9.84%), and hydrocarbons (9.29%), but of particular relevance were three major compounds identified with the highest relative peak-area percentages, which were 2,5-dimethyl-pyrazine (RT 31.12), benzaldehyde (RT 40.59), and cyclododecane (RT 70.55) (Figure 11). When the VOCs were analyzed at 4 h of bacterial growth (lag phase), the chemical compounds identified included ketones (12.85%), pyrazines (10.06%), and alcohols (8.94%). Interestingly, we detected new major compounds with the highest relative peak-area percentages: isopropyl alcohol (RT 9.63), ethanol (RT 9.89), and acetoin (RT 29.63). The percentages of 2,5-dimethyl-pyrazine (RT 31.12) and cyclododecane (RT 70.53) were maintained (Figure 11). Finally, the VOCs identified at 6 h of bacterial growth (stationary phase) included chemical classes of alcohols (13.07%), ketones (9.15%), and esters (7.84%). The major compounds detected with the highest relative peak-area percentages were ethanol (RT 9.90), acetoin (RT 29.62), and 2,5-dimethyl-pyrazine (TR 31.10), while the new major compounds detected at the highest relative peak-area percentages were 2,3-butanediol (RT 42.56) and 4,6-dichloro-5-cyanopyrimidine (RT 76.15) (Figure 11). Our results reveal that these VOCs produced during bacterial growth phases may be essential given their effect on *P. aroidearum* SM2 colonization and their capacity to reduce soft rot symptoms. Therefore, we decided to assess whether commercially available 2,5-dimethyl-pyrazine and acetoin impact *P. aroidearum* SM2 colonization on serrano chili fruits and reduce the presence of soft rot symptoms.

### 3.7. Evaluation of Pathogenic Inhibition by Standard VOCs

**Figure 12:**
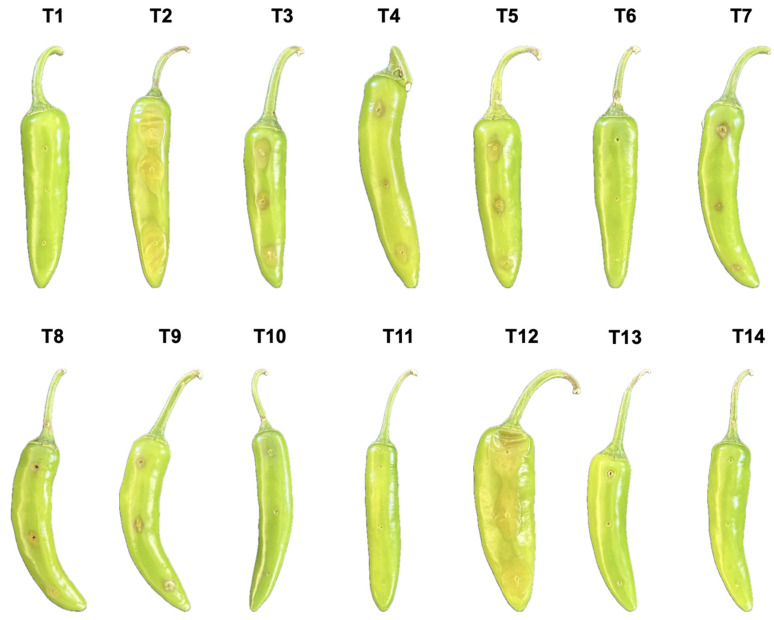
Evaluation of pathogenic inhibition by standard VOCs. In a total volume of 100 µL of sterile water, we resuspended both bacterial strains (10^8^ CFU/mL) for treatment without VOCs (T3) or with VOCs in addition to 5, 10, and 20 µL of 2,5-dimethyl-pyrazine (T4, T5, and T6) or acetoin (T7, T8, and T9) and a mixture of both VOCs at a volume concertation of 5 µL each (T10). T11 and T12 treatments only used *P. aroidearum* SM2 combined with 20 µL of 2,5-dimethyl-pyrazine or 20 µL of acetoin, respectively. The controls used sterile water (T1), *P. aroidearum* SM2 (T2), 2,5-dimethyl-pyrazine (T13), and acetoin (T14).

We decided to evaluate different volume concentrations of 2,5-dimethyl-pyrazine and acetoin independently and mixed, and we assessed them in relation to *K. cowanii* Cp1 to determine whether these chemical compounds are essential to colonization competition and the reduction in soft rot symptoms caused by *P. aroidearum* SM2 in serrano chili. In the results (Figure 12), 2,5-dimethyl-pyrazine at a volume of 20 µL achieved the complete inhibition of soft rot symptoms in treatments T6 and T11, while lower volumes (T4 and T5) yielded similar results to treatment T3, that is, reduced soft rot symptoms compared with the positive control (T2). When acetoin was tested, at all volume concentrations (T7, T8, and T9), the soft rot symptoms were less prevalent than those present in treatment T3, but when both VOCs were mixed at a volume of 5 µL each (T11), a complete inhibition of soft rot symptoms was observed. Interestingly, when *P. aroidearum* SM2 was tested with acetoin alone at a volume concentration of 20 µL (T12), the soft rot symptoms were similar to those seen in the positive control (T2). Negative controls did not show any perturbation (T1, T13, and T14). Additionally, bactericidal effects were observed on both bacterial strains when using 2,5-dimethyl-pyrazine at a volume concentration of 20 µL, with a zone of inhibition area of 2 mm. For acetoin, no effects were observed for any volume concentration tested. These results support the hypothesis that during competitive colonization, *K. cowanii* Cp1 synthetizes a mixture of VOCs to yield a competitive advantage.

## 4. Discussion

The phytopathogen *Pectobacterium* spp. is one of the most widely studied bacterial genera because it produces a significantly destructive disease in a wide diversity of plants around the world, causing significant business losses [7]. *P. aroidearum* was proposed as a novel bacterial species in 2013 and was described as a pathogen principally causing soft rot diseases in monocotyledonous plants [41,42]. Therefore, in this work, we report on the pathogen *P. aroidearum* SM2 isolated from the fruits of *C. annuum* var. Serrano with soft rot symptoms. Our genome sequencing analysis of *P. aroidearum* SM2 revealed important virulence genes that could be important during the maceration of the tissues of the fruits tested (*C. annuum* L., *Solanum lycopersicum*, *Physalis philadelphica*, and tubers of *Solanum tuberosum* (Figure 1)), such as pectinases, five types of secretion systems (type I, II, III, IV, and VI), and genes related to invasion motility (Table 3). In addition, according to the six antibiotic resistance mechanisms, the only phenotype showing resistance to erythromycin could potentially be accounted for via its genes related to efflux pump antibiotic resistance mechanisms. Previous research works showed that major pathogenicity determinants causing soft rot symptoms in the tissues of diverse economic crop plants include a battery of genes encoding extracellular pectinases, such as pectin lyase, pectin methylesterase, and pectin polygalacturonase, as well as proteinases, cellulases, toxins, and secretion systems, which are widely distributed and conserved in the genomes found in one of the top ten most common bacterial plant pathogens, *Pectobacterium* spp. [41,43,44].

Although diverse biocontrol strategies have been developed and employed to prevent the growth of *Pectobacterium* spp. and the plant diseases caused by it [1,2,3,4,5,6,7,8,9,10,11,12,13,14,15,16,17,18,19,20,21,22,23], additional investigations will be necessary into beneficial microorganisms that might compete successfully with this phytopathogen in an ecologically friendly manner, and thus help to reduce the environmental impacts on agriculture systems caused by the use of chemical bactericides [13,14,15]. In this regard, with reference to the hypotheses established in this work, the findings reported here demonstrate for the first time that *K. cowanii* Cp1, previously isolated from the seeds of *Capsicum pubescens*, can compete in a similar ecological niche against *P. aroidearum* SM2, leading to a reduction in soft rot symptoms in serrano chili (*Capsicum annuum* L.) and other vegetables, such as *Solanum lycopersicum*. Our results prove that if *K. cowanii* Cp1 colonizes the fruit tissue first, there is a high probability that it will reduce soft rot symptoms through the synthesis of a mix of VOCs, where alcohols, ketones, esters, pyrazines, and hydrocarbons can be important. Of the VOCs identified during the lag growth phase of *K. cowanii* Cp1, we have determined that 2,5-dimethyl-pyrazine is a chemical compound with bactericidal effects on *P. aroidearum* SM2 when applied at high volume concentrations, causing the complete elimination of soft rot symptoms. Interestingly, when 2,5-dimethyl-pyrazine was mixed with acetoin (3-hydroxy-2-butanone), identified during log and stationary growth phases, both VOCs showed synergy with *K. cowanii* Cp1 at lower volume concentrations, causing the complete elimination of soft rot symptoms compared with both VOCs tested separately at lower concentrations. These significant results require further investigation in order to obtain more information about the mechanisms of competitive colonization and interaction in the additional VOCs detected, and to evaluate potential chemical formulations that can act in synergy with *K. cowanii* Cp1 to more effectively control the phytopathogen *Pectobacterium* spp., as well as to possibly explore other microorganism pathogens.

The mechanisms by which bacterial VOCs alter plant pathogen growth and virulence represent an area that is attracting increasing interest [45,46,47]. In this regard, some studies have assessed how VOCs can also regulate bacterial virulence on *Pectobacterium* spp. during the course of pathogenesis [48]. In this context, the 2,3-butanediol pathway has been detected in *P. carotovorum* subsp. *carotovorum*, showing high gene expression (*budAB* operon) during pathogenesis in potato tubers, and *budB* gene disruption has been shown to lead to significantly less macerated tissue, but not growth inhibition [48]. Interestingly, the *budB* gene encoding an α-acetolactate synthase and participating in the condensation of two molecules of pyruvate into α-acetolactate, which are then converted into acetoin, has been associated with the alkalization of the growth culture medium, which favors pectate lyase activity in different microorganisms causing plant tissue disease [48]. Aligning with these reports, our results regarding treatment T12 encourage similar interpretations; in this treatment, acetoin was added and yielded marked soft rot symptoms, similar to the positive control treatment T2. However, regarding treatment T10, it remains to be explored as a mixture of acetoin and 2,5-dimethyl-pyrazine at lower volume concentrations can reduce soft rot symptoms.

These combined results suggest that a fluctuation in the relative concentrations of VOCs during competitive bacterial colonization affects pathogenic responses. A possible explanation of these results offered previously is that the diffusible quorum-sensing signal molecule N-acyl-homoserine lactone (AHL) could be affected by pH during the synthesis of acetoin, because it is hydrolyzed into an inactive form when above pH 6.8, thus affecting the regulation of key virulence genes’ expression [48,49]. Another possibility is that 2,5-dimethyl-pyrazine, which has been previously reported in other bacterial genera, and which has antifungal and antibacterial properties, could have metabolic effects at lower concentrations but bactericidal effects at higher concentrations [47]. On the other hand, although other individual VOCs identified in the lag growth phase were not tested, organic compounds such as cyclododecane could represent an area of interest, because this is a chemical compound with high toxicity that is persistent in the environment and has lower biodegradability [50]. Given its lipophilic nature, cyclododecane is easily absorbed and bioaccumulated in the fatty tissues of living organisms, and its growth toxicity in relation to bacteria has been evaluated [50]. Benzaldehyde is another chemical compound that is detected in the lag growth phase of *K. cowanii* Cp1; it is considered to be environmentally safe because it is highly biodegradable and presents bactericidal effects causing alterations in the cell membrane [51]. Therefore, the ways in which the properties of VOCs in combination or alone promote growth and pathogenic alterations in *P. aroidearum* SM2 require additional research.

Although some common chemical classes of VOCs detected in *K. cowanii* Cp1 have been identified in phylogenetically different bacterial species using single or mixed bacterial cultures [46,47], the fact that similar metabolic pathways are genetically conserved to produce VOCs suggests that similar physiological responses with different effects on competitive colonization can converge to enable efficient communication and antagonistic interactions between bacterial communities in a specific ecological niche. In this sense, the use of VOCs to enact the biocontrol of pathogenic microorganisms or to elicit different physiological plant responses must be pursued carefully to avoid undesired responses. For example, 2,3-butanediol can have negative impacts on bacterial growth, biofilm formation, and motility, but it can also promote pathogen resistance in plants [46,47]. 2,5-dimethyl-pyrazine is another common VOC produced by diverse bacterial species with fungal growth-inhibition effects [52], but it also regulates the gene expression involved in plant defense responses [47] and with bactericidal effects in significant plant pathogens such as *Ralstonia solanacearum* and *Xanthomonas axonopodis* pv. *punicae* [45,52]. Acetoin is synthetized by diverse microorganisms and has antifungal properties and promotes plant growth [46,47]. Therefore, it is questionable whether the VOCs produced by *K. cowanii* Cp1 play a specific role in its competitive colonization strategy against *P. aroidearum* SM2. In addition, it is possible that VOCs could have synergistic effects with other non-volatile antimicrobial compounds not detected in this work. With regard to this, diverse colonization strategies using biomolecules as antagonistic weapons have been detected in a wide variety of microorganisms [53]. For instance, 2,4-diacetylphloroglucinol (DAPG) and pyoverdine are relevant antimicrobial compounds produced by diverse *Pseudomonas* strains with a significant capacity to influence the structure of bacterial soil communities [53,54]. The production of peptide antibiotics, such as Pantocin A and B, has been identified in *Pantoea* sp.; these are used as biological control molecules against *P. carotovorum subsp. carotovorum* [55]. Therefore, research on these issues in relation to *K. cowanii* Cp1 is currently being undertaken so as to understand its ecological potential.

## 5. Conclusions

In this work, we reported the phytopathogen *P. aroidearum* SM2 with the ability to cause soft rot in the fruits of *C. annuum* L., *Solanum lycopersicum*, *Physalis philadelphica*, and the tubers of *Solanum tuberosum.* The competitive colonization results showed that a complex volatile mixture produced by *K. cowanii* Cp1 could probably be responsible in part for the reduction in soft rot caused by *P. aroidearum* SM2 as a competitive colonization strategy. Therefore, as the evidence suggests, *K. cowanii* Cp1 could play an essential ecological role during its interaction with plants to protect microorganism pathogens and represent a high potential bacterial strain to be applied in agriculture systems. In addition, given these notable results, it could be useful to assess how the signal transduction pathways which operate during plant–bacteria interactions are regulated in order to help us recognize beneficial bacterial species active during competitive colonization.

## Figures and Tables

**Table 1:** Competitive bacterial colonization interactions of *K. cowanii* Cp1 and *P. aroidearum* SM2.

Treatment	0 h	3 h	6 h	9 h
T1	*K. cowanii* Cp1/*P. aroidearum* SM2	N	N	N
T2	*P. aroidearum* SM2	*K. cowanii* Cp1	N	N
T3	*P. aroidearum* SM2	N	*K. cowanii* Cp1	N
T4	*K. cowanii* Cp1	*P. aroidearum* SM2	N	N
T5	*K. cowanii* Cp1	N	*P. aroidearum* SM2	N
T6	*K. cowanii* Cp1	N	N	*P. aroidearum* SM2
T7 *	*P. aroidearum* SM2	N	N	N
T8 *	*K. cowanii* Cp1	N	N	N
T9 *	0.1% peptone solution	N	N	N

* The controls were treatments 7, 8, and 9. N: not applicable.

**Table 2:** Main characteristics of the genome of *P. aroidearum* SM2.

Characteristics	Source	Chromosome
Genome length	PATRIC	5,037,920 bp
Number of contigs	PATRIC	86
Number of proteins characterized	PATRIC	3806
Number of putative/hypothetical proteins	PATRIC	1119
Number of rRNA genes	PATRIC	4
Number of tRNA genes	PATRIC	69
Number of proteins with pathway annotation	KEGG	789
G + C %	PATRIC	51.46%
N50 contig size (bp)	PATRIC	188,251 bp
Virulence factors	Victors	81
Virulence factors	VFDB	17
Virulence factors	PATRIC_VF	49
Transporter genes	TCDB	182
Drug target	DrugBank	154
Drug target	TTD	31
Antibiotic resistance	PATRIC	42
Antibiotic resistance	CARD	19

**Table 3:** Pathogenicity-related genes of *P. aroidearum* SM2 identified through gene annotation screening.

Type		Number	Gene
PCDEWs	Pectinases	12	*kgdM*, *exuT*, *uxaA*, *kdgF*, *ogl*, *pnl*, *exuR*, *pemA*, *pelW*, *pehX*, *pehT*, *pelX*
	Cellulases	0	
	Proteinases	0	
Secretion Systems	T1SS	22	*lapE*, *tonB*, *hlyA*, *tolC*, *hlyC*, *hlyB*, *hlyD*, *cyaB*, *cyaD*, *cyaE*, *hasE*, *lapC*, *prtE/aprE*, *lapD*, *prtD*/*aprD*, *lapB*, *lapG*, *tdfF*, *tdfG*, *tdfH*, *prtF/AprF*, *lnh*/*omp19*
	T2SS	22	*gspD*, *gspE*, *gspK*, *gspM*, *gspN*, *tadB*, *gspC*, *gspI*, *tadZ/cpaE*, *cpaD*, *flp/tadC*, *RcpC*/*CpaB*, *gspF*, *gspJ*, *gspL*, *gspB*, *gspG*, *gspH*, *tadD*, *cpaF*, *rcpA/cpaC*, *tadV*/*cpaA*
	T3SS	40	*yscN*, *mxiB*, *hroN*, *escN*, *hroT*, *yscR*, *hroR*, *escR*, *hrpQ*, *lcrE*, *yopN*, *yop4b*, *yscJ*, *hrcJ*, *escJ*, *pscJ*, *hrpW*, *yscQ*, *yscL*, *yscC*, *mixD*, *hrcC*, *invG*, *yscO*, *hrpB*, *hrpG*, *avrE1*, *hrcT*, *epaR1*, *escT*, *yscS*, *hrpV*, *yscU*, *lcrD*, *hrcV*, *escV*, *ssaV*, *yopN*, *icrE*, *hrpD*
	T4SS	19	*aroB*, *virB11*, *virD4*, *virB4*, *virB8*, *virB10*, *virB6*, *pilD*, *virB2*, *virB5*, *vir9*, *virB1*, *pilC*, *pilB*, *ppdD*, *pilM*, *pilN*, *pilP*, *pilQ*
	T5SS	0	
	T6SS	20	*paar*, *hcp*, *vgrG*, *tssI*, *rhaS*, *vasL*, *vasH*, *tssG*, *tssB*, *icmH*, *tssF*, *tssE*, *vasI*, *tssH*, *tssJ*, *tssA*, *tssK*, *tssM*, *tagH*, *tssC*
	Invasion motility	16	*fliN*, *fliA*, *cheR*, *fliM*, *flhD*, *flgC*, *flgG*, *fliP*, *fliG*, *fliS*, *flgH*, *flhC*, *cheW*, *cheY*, *flgB*, *fliQ*

## Data Availability

The data presented in this work are available from the corresponding authors upon request.

## Supplementary Materials

The following supporting information can be downloaded at: https://www.mdpi.com/article/10.3390/microorganisms12050930/s1, Figure S1: Two-compartment Petri dish plate device. On the bottom side of device *K. cowanii* Cp1 was inoculated on TSA medium and grown to produce any VOCs; Figure S2: Pathogenic inhibition assays using cell-free filtrate on chili fruits; Figure S3: Infection test of SM2 isolates on leaves of chili plants; Figure S4: Minimum inoculum of *P. aroidearum* SM2 causing soft rot symptoms; Figure S5: Detection of VOCs produced by *K. cowanii* Cp1; Table S1: Pairwise comparison of Average Nucleotide Identity (ANI) for *Pectobacterium* species; Table S2: Identification of VOCs produced by *K. cowanii* Cp1 at 2 h by HS-SPME-GC-MS; Table S3: Identification of VOCs produced by *K. cowanii* Cp1 at 4 h by HS-SPME-GC-MS; Table S4: Identification of COVs produced by *K. cowanii* Cp1 at 6 h p1 by HS-SPME-GC-MS.
