# Supplementary material for: Role of Volatile Organic Compounds Produced by Kosakonia cowanii Cp1 during Competitive Colonization Interaction against Pectobacterium aroidearum SM2
Source: Microorganisms. 2024 May 3;12(5):930. doi: 10.3390/microorganisms12050930 (PMC11123878; doi:10.3390/microorganisms12050930)
Supplement: Supplementary file 1 [file microorganisms-12-00930-s001.zip › microorganisms-2992201-supplementary.pdf]

microorganisms

Supplementary Results

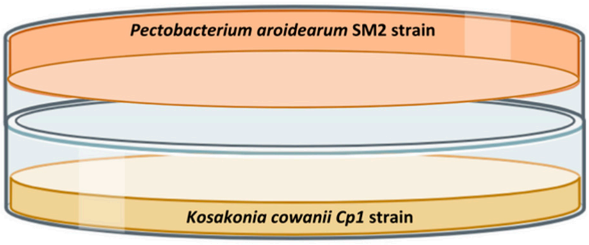

Figure S1. Two-compartment Petri dish plate device. On the bottom side of device *K. cowanii* Cp1 was inoculated on TSA medium and grown to produce any VOCs. *P. aroidearum* SM2 was inoculated on TSA medium (upper side) to evaluate colony growth inhibition.

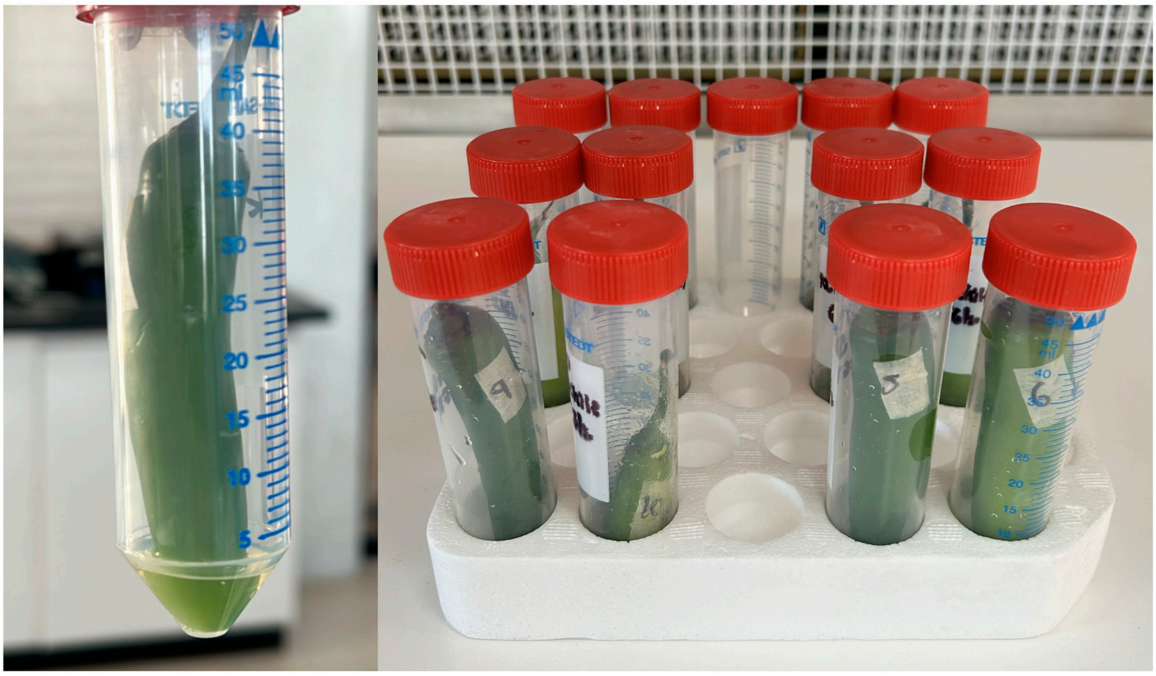

Figure S2. Pathogenic inhibition assays using cell-free filtrate on chili fruits.

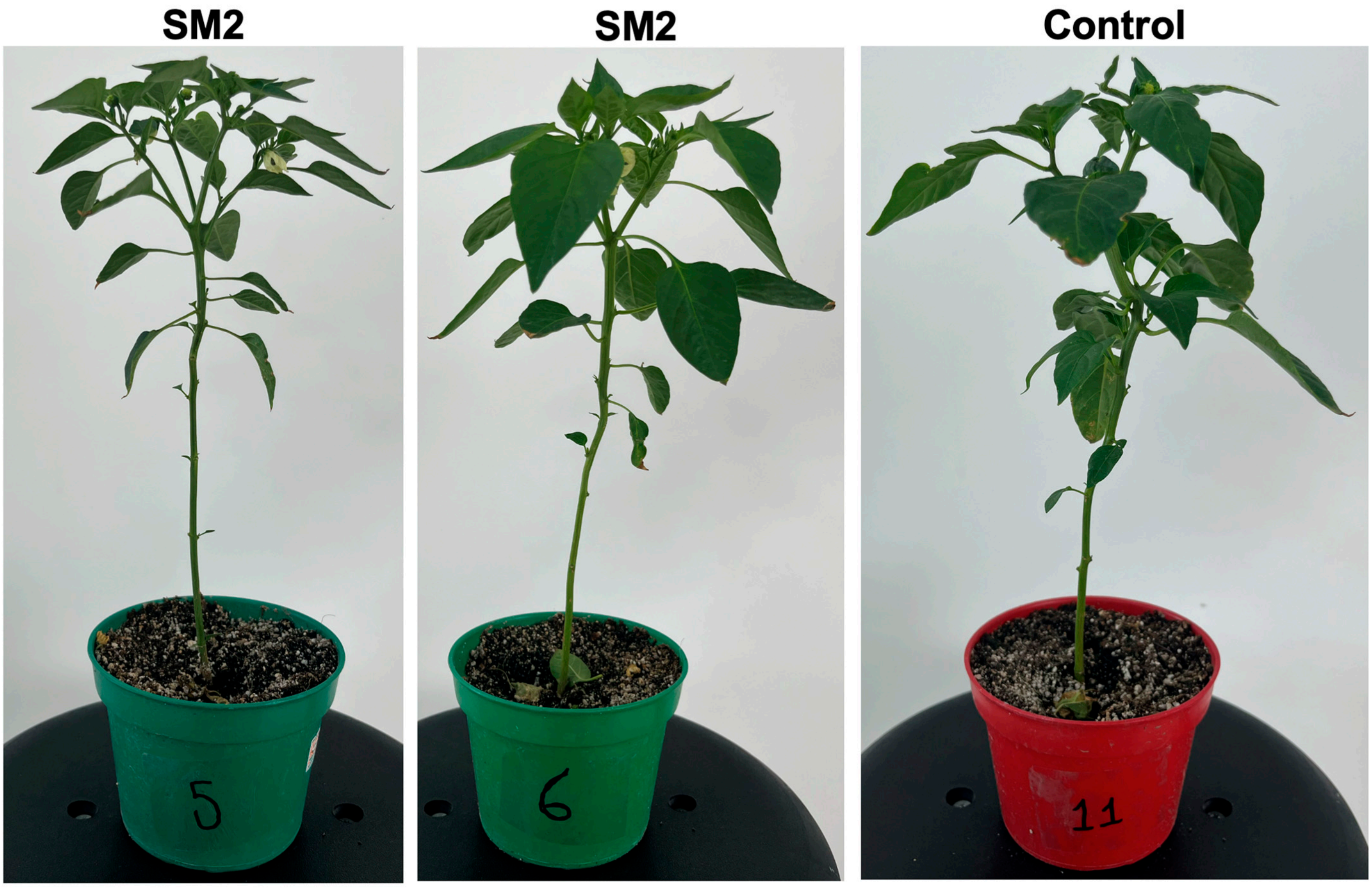

Figure S3. Infection test of SM2 isolates on leaves of chili plants.

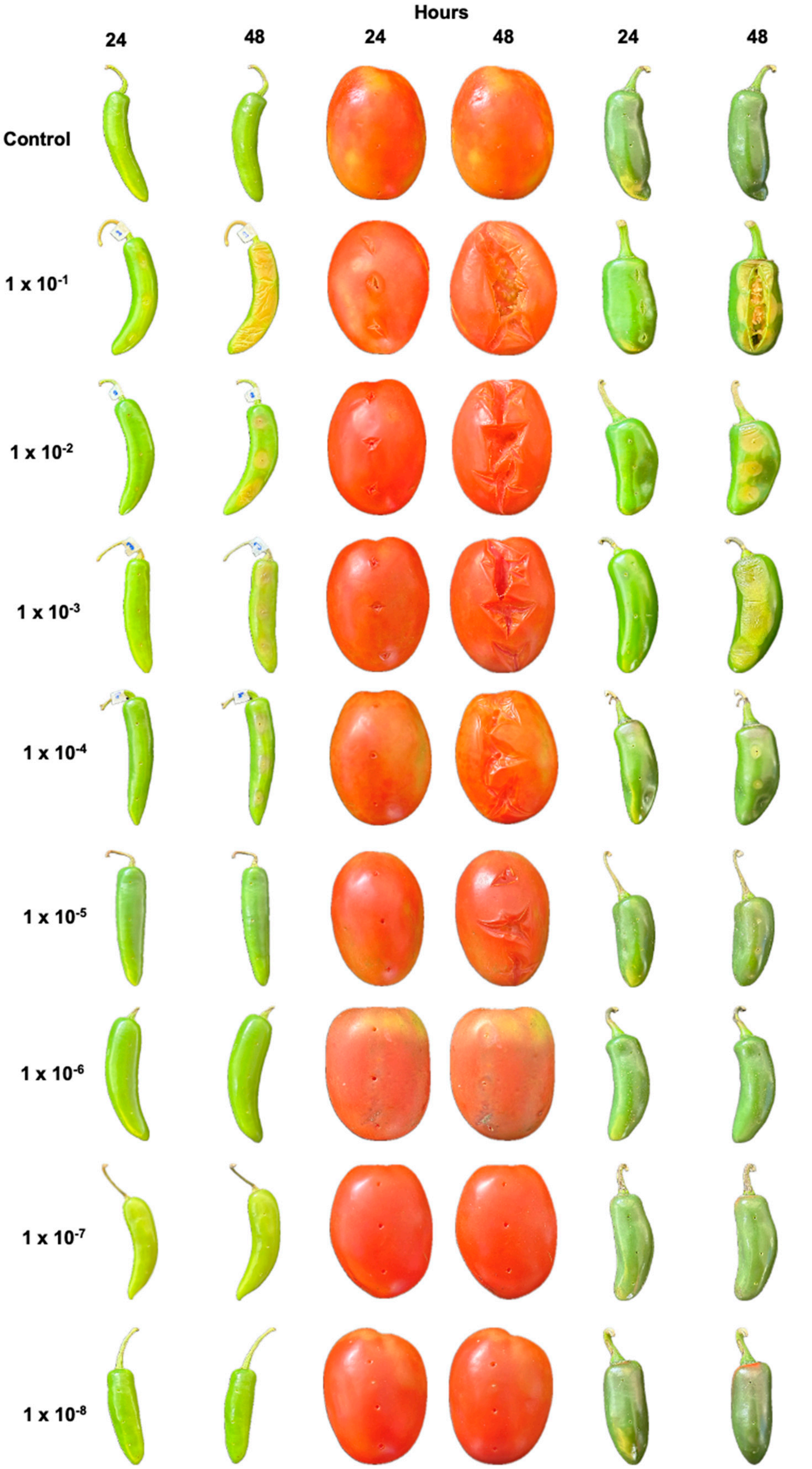

Figure S4. Minimum inoculum of *P. aroidearum* SM2 causing soft rot symptoms. Serial dilutions were made in 0.1% peptone and 10 µL bacterial suspensions were inoculated in each of the wounds, which were then incubated and evaluated for 48 hours.

Table S1. Pairwise comparison of Average Nucleotide Identity (ANI) for *Pectobacterium* species. ANI values ≥ 95% are colored red.

ANI VALUES ≥ 95%	*P. aroidearum* SM2	*P. aroidearum* L6	*P. aroidearum* QJ002	*P. actinidiae* KKH3	*P. aquaticum* A212-S19-A16	*P. atrosepticum* NCPPB 549	*P. betavasculorum* NCPPB 2795	*P. brasiliense* LMG 21371	*P. carotovorum* DSM 30168	*P. colocasium* LJ1	*P. fontis* M022	*P. jejuense* 13-115	*P. odoriferum* NCPPB 3839	*P. parmentieri* RNS 08-42-1A	*P. parvum* s0421	*P. peruviense* IFB5232	*P. polaris* NIBIO1006	*P. polonicum* DPMP315	*P. punjabense* SS95
P. aroidearum SM2		97.30	97.25	89.21	89.34	88.29	88.29	90.18	89.93	90.56	87.48	92.79	92.99	88.28	92.92	88.21	93.20	89.34	88.55
P. aroidearum L6	97.16		97.81	89.22	89.45	88.44	88.39	90.21	89.79	90.55	87.44	90.14	89.46	88.01	88.66	88.60	89.88	88.51	88.44
P. aroidearum QJ002	97.26	97.98		89.25	89.58	88.59	88.29	90.25	89.93	90.56	87.47	90.31	89.58	88.01	89.74	88.66	89.92	88.53	88.48
P. actinidiae KKH3	88.96	89.10	88.98		91.18	88.57	88.15	90.72	92.55	89.23	87.62	91.07	92.01	87.63	90.86	88.58	91.02	88.67	87.79
P. aquaticum A212-S19-A16	89.52	88.56	89.70	91.58		89.04	88.77	92.80	93.67	89.48	87.79	92.79	92.99	88.28	92.92	89.21	93.20	89.34	88.55
P. atrosepticum NCPPB 549	88.39	88.56	88.58	88.76	89.00		91.25	89.23	89.36	88.07	87.19	89.18	88.91	89.46	89.27	93.59	89.42	89.19	89.12
P. betavasculorum NCPPB 2795	88.40	88.50	88.32	88.35	88.74	91.39		88.73	88.97	87.79	86.85	88.90	88.54	88.53	88.86	91.69	89.07	88.67	88.64
P. brasiliense LMG 21371	90.05	90.27	90.18	90.89	92.54	89.11	88.54		92.68	89.67	87.58	94.17	91.34	88.32	93.08	89.15	93.46	88.91	88.67
P. carotovorum DSM 30168	89.88	89.87	89.80	92.70	93.47	89.28	88.81	92.77		89.85	87.83	93.30	94.81	88.30	92.87	89.39	93.10	89.84	88.60
P. colocasium LJ1	90.48	90.58	90.53	89.32	89.38	87.99	87.56	89.71	89.83		87.50	89.92	89.44	87.42	89.51	87.97	89.70	88.15	87.63
P. fontis M022	87.60	87.69	87.66	87.97	87.76	87.27	86.91	87.75	88.09	87.76		87.98	88.02	86.64	87.94	87.30	87.89	87.34	86.79
P. jejuense 13-115	89.98	90.17	90.18	91.16	92.45	88.92	88.70	94.02	93.20	89.88	87.68		91.95	88.24	93.82	89.03	94.63	89.14	88.40
P. odoriferum NCPPB 3839	89.34	89.49	89.45	92.26	92.74	89.03	88.65	91.61	94.68	89.46	87.88	92.21		88.04	92.31	89.16	92.32	89.59	88.47
P. parmentieri RNS 08-42-1A	87.76	88.03	87.94	87.84	88.10	89.40	88.53	88.29	88.33	87.44	86.48	88.23	88.13		88.32	89.49	88.36	90.60	91.06
P. parvum s0421	89.81	88.89	89.87	91.25	92.93	89.33	88.77	93.19	93.01	89.67	87.91	94.04	92.53	88.52		89.38	95.92	89.29	88.71
P. peruviense IFB5232	88.35	88.61	88.59	88.67	89.05	93.55	91.51	89.08	89.29	88.03	87.01	89.13	89.13	89.44	89.17		89.24	89.16	89.20
P. polaris NIBIO1006	89.92	89.86	89.91	91.29	93.06	89.34	88.98	93.57	93.20	89.82	87.71	94.73	92.46	88.34	95.70	89.34		89.20	88.71
P. polonicum DPMP315	88.27	88.47	88.42	88.83	89.09	89.13	88.49	88.91	89.88	88.12	87.06	89.11	89.58	90.63	89.00	89.20	89.12		93.54
P. punjabense SS95	88.32	88.43	88.37	87.94	88.33	89.11	88.50	88.70	88.62	87.60	86.62	88.44	88.39	91.03	88.46	89.20	88.66	93.51	

Table S2. Identification of VOCs produced by *K. cowanii* Cp1 at 2 h by HS-SPME-GC-MS.

Compounds	Retention Time (min)	Relative Peak Area (%)	Chemical Classes	Compounds	Retention Time (min)	Relative Peak Area (%)	Chemical Classes
Carbon dioxide	4.219	1.23	Hydrocarbon	Piconol	44.999	0.09	Other compounds
Semicarbazide	4.467	0.5	Amide	2-Butyl-3-methylpyrazine	45.26	0.08	Pyrazines
n-Hexane	4.578	0.28	Hydrocarbon	Benzeneacetaldehyde	45.728	0.52	Aldehydes
Ethyl ether	4.636	0.88	Ether	2-Acetylthiazole	45.911	0.36	Ketones
Methanethiol	4.895	0.53	Tioles	Acetophenone	46.062	0.48	Ketones
Heptane	5.093	1.82	Hydrocarbon s	2-Furanmethanol	46.38	1	Aromatic compounds
Acetone	6.39	0.94	Ketones	Pyrazine, 2-butyl-3,5-dimethyl-	46.729	0.62	Pyrazine
Cyclotrisiloxane, hexamethyl-	7.435	2.79	Organosilico s compounds	1,3-Oxathiolane, 2-methyl-2-phenyl-	47.133	0.08	Esters
2-Propanone, hydrazone	7.793	1.14	Ketone	Benzaldehyde, 2-hydroxy-	47.757	0.16	Aldehydes
2-Butanone	8.539	1.15	Ketone	4-Carene, (1S,3S,6R)-(-)-	48.514	0.04	Hydrocarbons
Butanal, 2-methyl-	9.059	0.42	Aldehyde	Silane, diethyldi(3-phenylpropoxy)-	48.82	0.05	Other compounds
Butanal, 3-methyl-	9.25	2.5	Aldehyde	3,4-Dihydro-1-methylpyrrolo[1,2-a]pyrazine	49.067	0.12	Pyrazine
Ethanol	9.83	0.04	Alcohol	2-Furanmethanol, 5-methyl-	49.611	0.06	Alcohols
2,3-Butanedione	12.5	0.48	Ketone	Tridecanal	50.032	0.49	Aldehyde
Trichloroethylene	13.038	0.33	Hidrocarbon s	Cyclopenten-4-one, 1,2,3,3-tetramethyl-	51.604	1	Hydrocarbons
Acetonitrile	13.59	0.03	Other compounds	R-Palmitoyl-(1-methyl) ethanolamide	51.851	0.04	Amide
Methyl Isobutyl Ketone	13.708	0.05	Ketone	Cyclopropane, octyl-	52.556	0.26	Hydrocarbons
Cyclotetrasiloxane, octamethyl-	14.283	2.51	Organosilico s compounds	7-Chloro-2,3-dihydro-3-(4-N,N-dimethylaminobenzylidene)-5-phenyl-1H-1,4-benzodiazepin-2-one	55.995	0.09	Other compounds
Trichloromethane	14.768	0.51	Other compounds	2-Tridecanone	56.873	0.19	Ketones
Ethyl 3-amino-4-(1,3-benzodiazol-1-yl)benzoate	15.131	0.13	Esther	Cyclodecene	58.078	0.05	Alkenes
Toluene	15.417	0.14	Hydrocarbon s	Cyclononasiloxane, octadecamethyl-	58.314	0.24	Organosilicos compounds
Disulfide, dimethyl	17.138	0.42	Other compounds	Cinnoline, 3-methyl-	59.162	0.08	Aromatic compounds
N-(4-Methylbenzenesulfonyl)-2-methylazetidin-3-one	17.749	0.12	Ketones	Benzyl alcohol	60.946	1.02	Alcohols
Hexanal	18.077	0.14	Aldehydes	Butanoic acid, butyl ester	61.691	0.37	Acids
1,2-Dichloroethyl hydroperoxide	18.231	0.02	Esthers	2,2,4-Trimethyl-1,3-pentanediol diisobutyrate	62.951	0.15	Alcohols
8-Nonynoic acid	18.342	0.1	Acids	n-Decanoic acid	63.669	0.22	Acids
2-Butenal, 2-methyl-, (E)-	18.669	0.33	Aldehydes	Benzenepentanamide	64.029	0.09	Acids
Acetic acid, chloro-	18.973	0.08	Acids	Phenylethyl Alcohol	64.415	0.07	Alcohols
o-Xylene	20.23	0.04	Hydrocarbon s	Diethylmalonic acid, 2,2,3,3,4,4,4-heptafluorobutyl hexyl ester	64.76	0.16	Esters
p-Xylene	20.739	0.14	Hydrocarbon s	Benzene, 1-isocyano-2-methyl-	65.925	0.04	Other compounds
3-Penten-2-one, 4-methyl-	21.039	0.29	Ketones	2-Butanone, 1,1-dichloro-3,3-dimethyl-	67.385	0.09	Ketones
1-Butanol	22.042	0.28	Alcohols	S-Methyl 3-methylbutanethioate	69.092	0.11	Esters
Nonane	22.549	0.05	Hydrocarbon s	Ethanone, 1-(1H-pyrrol-2-yl)-	69.372	0.04	Ketones
Pyridine	23.463	0.03	Aromatic compounds	Cyclododecane	70.558	13.44	Hydrocarbons

Cyclopentasiloxane, decamethyl-	23.812	0.6	Organosilicos compounds	Trimethylsilyl [2-(4-chlorophenyl)-4-phenyl-1,3-thiazol-5-yl]acetate	71.595	0.22	Other compounds
Cyclopentasiloxane, decamethyl-	23.904	1.15	Organosilicos compounds	2-Pyrrolidinone	71.854	0.18	Other compounds
2-Butenal, 3-methyl-	24.775	0.1	Aldehydes	Phenol	72.061	0.36	Aromatics compounds
D-Limonene	25.187	0.07	Hydrocarbons	1,2,3,4-Tetrahydrofluorene	72.378	0.15	Hydrocarbons
Pyrazine	25.358	2.34	Pyrazines	Heptanoic acid	72.635	0.14	Acids
Phthalic acid, 2-(2-nitrophenyl)ethyl nonyl ester	25.755	0.04	Esters	Propane, 1-isothiocyanato-	72.697	0.17	Other compounds
Thiazole	27.312	0.07	Other compounds	1H,1H,2H,2H-Perfluorooctan-1-ol	72.889	0.22	Alcohols
Aziridine, 1-(2-phenylethyl)-	27.898	0.07	Other compounds	Cyclodecasiloxane, eicosamethyl-	73.228	0.28	Organosilicos compounds
Pyrazine, methyl-	28.213	2.17	Pyrazines	2-Propenal, 3-phenyl-	73.559	0.16	Aldehydes
Ethanone, 1-cyclopentyl-	28.781	0.03	Ketones	2-Pentadecanone	74.507	0.23	Ketones
Acetic acid, methoxy-, ethyl ester	29.631	0.11	Esters	Cyclohexanol, 5-methyl-2-(1-methylethyl)-, (1.alpha.,2.alpha.,5.alpha.)-	75.73	0.15	Alcohols
Octanal	29.991	0.07	Aldehydes	5-Methyl-2-phenyl-2-hexenal	76.243	0.3	Aldehydes
Carbonofluoridic acid, trifluoromethyl ester	30.094	0.05	Esters	p-Cresol	76.565	0.04	Aromatic compounds
2-Propanone, 1-hydroxy-	30.339	0.41	Ketones	2-Piperidinone	76.809	0.03	Other compounds
Pyrazine, 2,5-dimethyl-	31.125	19.06	Pyrazines	3-tert-Butyl-4-hydroxyanisole, acetate	78.349	0.05	Aromatic compounds
Pyrazine, 2,6-dimethyl-	31.442	0.7	Pyrazines	Octanoic acid	78.625	1.14	Acids
Pyrazine, ethyl-	31.781	0.13	Pyrazines	As-indacen-3-one, 1,1,8b-trimethyl-1,4,5,8b-tetrahydro-2-oxa-3a,7,8a-triaza-	79.345	0.04	Other compounds
Cyclohexasiloxane, dodecamethyl-	32.252	0.82	Organosilicos compounds	Valeric acid, 2,4,6-trichlorophenyl ester	79.524	0.08	Esters
Pentanoic acid, 4-methyl-, ethyl ester	32.574	0.13	Esters	4-Methylphenol, isopropyl ether	79.787	0.09	Ethers
Hexyl chloroformate	32.845	0.07	Other compounds	2,6-Dihydroxybenzoic acid, 3TMS derivative	80.568	0.17	Acids
(Dimethylaminomethylene)malononitrile	33.265	0.04	Other compounds	2H-Pyran-2-one, 6-pentyl-	80.936	0.12	Esters
n-Propyl t-butyl ether	33.338	0.06	Ethers	1H-Imidazole, 2-methyl-	81.376	0.05	Other compounds
Dimethyl trisulfide	33.973	0.18	Other compounds	Cyclododecane	81.927	0.09	Hydrocarbons
Pyrazine, 2-ethyl-6-methyl-	34.329	0.11	Pyrazines	5.alpha.-Androstan-1-methylene-3.alpha.-ol-17-one, di-trimethylsilyl	82.194	0.04	Other compounds
Pyrazine, 2-ethyl-5-methyl-	34.599	1.44	Pyrazines	5-Acetyl-2-methylpyridine	82.511	0.05	Other compounds
Benzenamine, N,N,2-trimethyl-	34.85	0.05	Amines	Cyclononasiloxane, octadecamethyl-	82.936	0.13	Organosilicos compounds
2-Nonanone	34.963	0.15	Ketones	Ethanone, 1-(4-ethylphenyl)-	83.089	0.14	Ketones
Pyrazine, trimethyl-	35.122	1.83	Pyrazines	Methyl anthranilate	83.531	3.22	Esters
Pyrazine, 2-methyl-5-(1-methylethyl)-	35.671	0.34	Pyrazines	Methanone, (7-bromo-2,3-dihydro-1,4-benzodioxin-6-yl)2-thienyl-	84.279	0.09	Ketones
Pyridine, 2-(1-methylethyl)-	36.659	0.12	Pyrazines	Benzamide	84.559	0.38	Amides
Pyrazine, 3-ethyl-2,5-dimethyl-	37.156	1.44	Pyrazines	Sorbic Acid	85.086	0.18	Acids
Isobutyl 4-methylpentan-2-yl carbonate	37.429	0.13	Other compounds	1-(4-Nitrophenyl)pyrazole-4-carboxylic acid	85.229	0.13	Acids
Methional	37.536	0.18	Hydrocarbons	Ethanol, 2,2-dichloro-	85.364	0.13	Alcohols
Isobutyl 2,5,8,11-tetraoxatridecan-13-yl carbonate	37.757	0.07	Other compounds	Benzonitrile, 2-hydroxy-	85.509	0.35	Other compounds

Pyrazine, 2-ethyl-3,5-dimethyl-	37.891	0.1	Pyrazines	Ethyl 5-methyl-1,3,4-oxadiazole-2-carboxylate	85.794	0.16	Aromatic compounds
Furfural	38.001	0.27	Other compounds	Cyclononasiloxane, octadecamethyl-	85.903	0.46	Organosilicos compounds
.alpha.-Terpineol	38.402	0.04	Other compounds	Sebacic acid, 2-hexyl isobutyl ester	86.487	0.3	Esters
4-Heptanol, 2,6-dimethyl-	38.586	0.16	Alcohols	Benzenemethanol, 2-hydroxy-5-methyl-	86.725	0.25	Alcohols
Pyrazine, 2-ethenyl-6-methyl-	39.055	0.09	Pyrazines	2,4-Di-tert-butylphenol	87.026	0.39	Aromatic compounds
1-Hexanol, 2-ethyl-	39.232	0.77	Alcohols	n-Decanoic acid	88.039	0.9	Acids
Cycloheptasiloxane, tetradecamethyl-	39.517	0.49	Alcohols	Diethyl Phthalate	88.843	0.15	Esters
Ethanone, 1-(2-furanyl)-	39.815	0.09	Ketones	3-Chloro-6-methyl-6,7-dihydro-9H-5-oxa-9-azabenzocyclohepten-8-one	89.48	0.08	Other compounds
2H-Pyran-2,5-diol, tetrahydro-, diacetate	40.06	0.07	Other compounds	1,3-Dioxolane, 4-ethyl-5-octyl-2,2-bis(trifluoromethyl)-, trans-	90.02	0.02	Other compounds
3-Phenylindole	40.21	0.41	Other compounds	Phenol, 4-(1,1-dimethylpropyl)-	90.378	0.04	Aromatic compounds
Benzaldehyde	40.596	5.38	Aldehydes	Tetracosamethyl-cyclododecasiloxane	91.188	0.02	Other compounds
2,4,4,6,6,8,8-heptamethyl-1,3,5,7,2,4,6,8-tetraoxatetrasilocan-2-ol	40.927	0.5	Other compounds	Benzene, 1-chloro-4-[2-nitro-1-(2-propenylthio)ethyl]-	92.854	0.05	Other compounds
Pyrazine, 2-methyl-3-(2-propenyl)-	41.242	0.6	Pyrazines	S-Methyl isopropylphosphonamidothioate	93.447	0	Other compounds
benzenamine, 3-[(tetrahydro-2H-pyran-2-yl)oxy]-	41.472	0.09	Other compounds	2-Methoxyethoxy-ethanol	93.647	0.01	Aromatic compounds
1-Octanol	42.082	0.18	Alcohols	3,5-di-tert-Butyl-4-hydroxybenzaldehyde	93.877	0.16	Aldehydes
Fluoren-9-ol, 3,6-dimethoxy-9-(2-phenylethynyl)-	42.628	0.24	Other compounds	4-Nitrophenyl trifluoromethanesulfonate	94.247	0	Aromatic compound
Phosphonoacetic Acid, 3TMS derivative	42.907	0.29	Acids	2-Trifluoromethylbenzoic acid, 4-methylpentyl ester	95.251	0.04	Esters
Pyrazine, 2-(n-propyl)-	43.167	0.17	Pyrazines	Cyclodecasiloxane, eicosamethyl-	95.392	0.03	Organosilicos compounds
Phosphonoacetic Acid, 3TMS derivative	43.547	0.14	Acids	6-Amino-2-(fluoromethylidene)-3-(2-methylphenyl)-2,3-dihydroquinazolin-4(1H)-one, mono(trimethylsilyl) derivative	95.481	0.03	Other compounds
Benzonitrile	44.114	0.05	Benzens	4-Hydroxy-2-methoxybenaldehyde	95.624	0.04	Aromatic compounds
2-Undecanone	44.293	0.21	Hydrocarbons	n-Hexadecanoic acid	95.865	0.15	Acids
Androsta-1,4-dien-3-one, 17-[(1-oxo-10-undecenyl)oxy]-, (17.beta.)-	44.413	0.07	Other compounds	Chloromethanesulfonyl chloride	96.396	0.01	Other compounds
Ethanol, 2-(2-ethoxyethoxy)-	44.529	0.11	Alcohols	Benzoic acid	96.755	0.03	Other compounds
				Phthalimide	97.679	0.14	Amide

Table S3. Identification of VOCs produced by *K. cowanii* Cp1 at 4 h by HS-SPME-GC-MS.

Compounds	Retention Time (min)	Relative Peak Area (%)	Chemical Classes	Compounds	Retention Time (min)	Relative Peak Area (%)	Chemical Classes
Carbon dioxide	4.2126	1.5782	Hydrocarbons	Silane, dimethyl(dimethyl(dimethyl(2-isopropylphenoxy)silyloxy)silyloxy)(2-isopropylphenoxy)-	42.9053	0.3848	Organosilicos compounds
2-Octanamine	4.3659	1.7024	Amines	2-Isoamylpyrazine	43.1543	0.3359	Pyrazines
Ethyl ether	4.6355	1.0973	Ethers	Phosphonoacetic Acid, 3TMS derivative	43.5387	0.1748	Acids
Methanethiol	4.8934	0.6788	Tioles	2-Undecanone	44.2764	0.4162	Ketones
Heptane	5.0895	3.2621	Hydrocarbons	Pyrazine, 3,5-dimethyl-2-propyl-	44.4327	0.2634	Pyrazines
Acetone	6.3956	1.2747	Ketones	2-Isoamyl-6-methylpyrazine	44.9965	0.1323	Pyrazines
Cyclotrisiloxane, hexamethyl-	7.4366	1.6261	Other compounds	Pyrazine, 2-methyl-5-propyl-	45.2461	0.1398	Pyrazines
Butanal	7.8132	0.5795	Aldehydes	Benzeneacetaldehyde	45.7213	0.5201	Aldehydes
(3-Methyl-oxiran-2-yl)-methanol	8.2514	0.2956	Alcohols	2-Acetylthiazole	45.9054	0.307	Tioles
2-Butanone	8.5563	1.0136	Ketones	Acetophenone	46.0589	0.2813	Ketones
Butanal, 2-methyl-	9.0824	0.3761	Aldehydes	N-(2-Acetylcyclohexylidene)cyclohexylamine	46.2014	0.0829	Other compounds
Butanal, 3-methyl-	9.2754	1.5231	Aldehydes	2-Furanmethanol	46.3717	0.6816	Alcohols
Isopropyl Alcohol	9.6344	7.9273	Alcohols	Pyrazine, 2,5-dimethyl-3-(3-methylbutyl)-	46.7182	1.5359	Pyrazines
Ethanol	9.8968	5.2798	Alcohols	Butanoic acid	47.1192	0.1874	Acids
2-Pentanone	12.0822	0.0405	Ketones	Propanedinitrile, (ethoxymethylene)-	47.7582	0.108	Other compounds
n-Propyl acetate	12.1995	0.0314	Esters	Benzoic acid, 4-methyl-	48.7692	0.0674	Acids
2,3-Butanedione	12.5333	0.6946	Ketones	1-Propanol, 3-(methylthio)-	49.061	0.1723	Alcohols
Trichloroethylene	13.0685	0.3244	Halocarbons	Butanoic acid, 3-methyl-	49.4532	0.0662	Acids
Acetonitrile	13.6221	0.0406	Other compounds	2-Furanmethanol, 5-methyl-	49.5847	0.0807	Alcohols
Methyl Isobutyl Ketone	13.7437	0.0564	Ketones	Tetradecanal	50.0238	0.3508	Aldehydes
Cyclotetrasiloxane, octamethyl-	14.3047	1.5744	Other compounds	Acetamide	50.7548	0.0706	Amidas
Trichloromethane	14.8041	0.2079	Other compounds	Benzene, 1,3-dimethoxy-	51.6009	0.0863	Esters
2-1-Phenyl ethylidene-hydrazono-3-methyl-2,3-dihydrobenzothiazole	15.0525	0.216	Other compounds	1-Decanol	52.5638	1.9182	Alcohols
Toluene	15.4514	0.2354	Hydrocarbons	Acetamide, N,N-dibutyl-	54.253	0.0595	Amidas
Dodecane	16.1144	0.0359	Hydrocarbons	10-Undecen-1-ol	56.403	0.2586	Alcohols
Disulfide, dimethyl	17.1678	1.1384	Other compounds	2-Tridecanone	56.8746	0.3131	Ketones
S-(Isobuthoxythiocarbonyl)thiohydroxylamine	17.7703	0.0724	Other compounds	Naphthalene, decahydro-, cis-	58.0396	0.5871	Hidrocarbons
Hexanal	18.1055	0.0836	Aldehydes	Furan, 3-phenyl-	59.1475	0.1969	Other compounds
Acetamide, N-2-propynyl-	18.3532	0.0765	Other compounds	Benzyl alcohol	60.9237	0.2226	Alcohols
2-Butenal, 2-methyl-	18.6806	0.1793	Other compounds	Butanoic acid, butyl ester	61.6643	0.3129	Esters
Ethylbenzene	20.325	0.0762	Hydrocarbons	Propanoic acid, 2-methyl-, 2-methylpropyl ester	62.9301	0.1483	Esters
p-Xylene	20.779	0.1378	Hydrocarbons	5-Methylhexanoic acid	63.578	0.2454	Acids
3-Penten-2-one, 4-methyl-	21.0413	0.0793	Ketones	Methoxycarbonyl methoxythioxomethyl disulfide	64.0157	0.0527	Other compounds
Benzene, 1,3-dimethyl-	21.2047	0.1547	Other compounds	Phenylethyl Alcohol	64.4037	0.143	Other compounds
1-Butanol	21.9719	0.5005	Alcohols	Benzonitrile, 2-methyl-	65.897	0.0309	Other compounds
3-Heptanone, 2-methyl-	22.6077	0.0286	Ketones	Benzene, (1-pentylheptyl)-	66.5935	0.1485	Esters
Boron, trihydro(pyridine)-, (T-4)-	23.4265	0.0469	Other compounds	2-Butanone, 1-chloro-3,3-dimethyl-	67.3667	0.0547	Ketones

Cyclopentasiloxane, decamethyl-	23.8904	1.2733	Organosilicos compounds	2,5-Piperazinedione, 3-methyl-	69.0691	0.041	Other compounds
2-Butenal, 3-methyl-	24.7753	0.046	Aldehydes	Ethanone, 1-(1H-pyrrol-2-yl)-	69.3625	0.0305	Ketones
D-Limonene	25.1624	0.0447	Other compounds	Cyclododecane	70.5381	7.014	Other compounds
1-Butanol, 3-methyl-	25.4302	3.0905	Alcohols	3-[5-(Chloromethyl)-1,2,4-oxadiazol-3-yl]-4-nitro-1,2,5-oxadiazole	71.5533	0.0482	Oxadiazoles
Benzene, 1-isothiocyanato-4-methyl-	25.7529	0.0224	Other compounds	2-Pyrrolidinone	71.8419	0.0555	Pyrrolidinones
Thiazole	27.3075	0.0532	Tioles	Phenol	72.0613	0.1484	Other compounds
l-Phenylalanine, methyl ester	27.4226	0.0279	Esters	1,2,3,4-Tetrahydrofluorene	72.3295	0.0297	Tetrahydrofluorenes
1,3,5,7-Cyclooctatetraene	27.9687	0.0571	Other compounds	Heptanoic acid	72.7211	0.1349	Other compounds
Pyrazine, methyl-	28.2136	1.5754	Pyrazines	Bicyclo[4.1.0]heptane, 2-methyl-	72.8892	0.1597	Ketones
Silanediol, dimethyl-, diacetate	28.8063	0.012	Other compounds	Cyclodecasiloxane, eicosamethyl-	73.2191	0.1941	Organosilicos compounds
2-fluoro-1-phenylethanone	29.3166	0.0483	Ketones	9-Dodecen-1-ol, acetate, (Z)-	73.934	0.213	Acids
Acetoin	29.6334	5.4583	Ketones	2-Nonadecanone	74.3816	0.1106	Ketones
Octanal	30.0745	0.1251	Aldehydes	Z-(13,14-Epoxy)tetradec-11-en-1-ol acetate	75.704	0.2352	Other compounds
2-Propanone, 1-hydroxy-	30.3204	0.2289	Ketones	5-Methyl-2-phenyl-2-hexenal	76.2341	0.1513	Other compounds
Pyrazine, 2,5-dimethyl-	31.1206	12.5776	Pyrazines	Phenol, 3-methyl-	76.5431	0.0353	Aromatic compounds
Pyrazine, 2,6-dimethyl-	31.4411	0.6237	Pyrazines	Tris(dimethylamino)borane	76.7715	0.0155	Other compounds
Pyrazine, ethyl-	31.786	0.1253	Pyrazines	Glycerol 1,2-diacetate	76.9813	0.0116	Other compounds
Cyclohexasiloxane, dodecamethyl-	32.2545	0.5663	Organosilicos compounds	N-(6-Methoxy-1,3-benzothiazol-2-yl)acetamide	78.3256	0.0248	Amidas
Heptanoic acid, ethyl ester	32.572	0.0804	Esters	Octanoic acid	78.7092	0.8449	Acids
Formic acid, hexyl ester	32.8456	0.1912	Esters	2-Trichloroacetylpyrrolidine	79.3402	0.0322	Other compounds
Hexane, 1,2,3-trimethoxy-	33.2567	0.0188	Hidrocarbons	2(3H)-Furanone, 5-heptyldihydro-	79.5124	0.0346	Other compounds
2-Pentanone, 4-hydroxy-4-methyl-	33.3481	0.0276	Ketones	4-[4-[(2-Hydroxybenzoyl)amino]anilino]-4-oxobut-2-enoic acid	79.7864	0.0477	Other compounds
Dimethyl trisulfide	34.0029	0.2624	Other compounds	Cyclodecasiloxane, eicosamethyl-	80.5595	0.0802	Organosilicos compounds
Pyrazine, 2-ethyl-6-methyl-	34.3283	0.1344	Pyrazines	2H-Pyran-2-one, 6-pentyl-	80.9195	0.0519	Ketones
Pyrazine, 2-ethyl-5-methyl-	34.6025	1.0039	Pyrazines	4,7-Methano-1H-indene, octahydro-, (3a.alpha.,4.alpha.,7.alpha.,7a.alpha.)-	81.0509	0.019	Hydrocarbons
2-Nonanone	34.9697	0.8794	Ketones	Cyclohexene, 3-(2-methylpropoxy)-	81.4896	0.1175	Hydrocarbons
Pyrazine, trimethyl-	35.1229	1.5173	Pyrazines	Cyclopentadecane	82.0705	0.2891	Ketones
Pyrazine, 2-methyl-5-(1-methylethyl)-	35.6756	0.2632	Pyrazines	Ethanone, 1-(2-aminophenyl)-	82.49	0.0432	Ketones
Benzenamine, 4-(2H-tetrazol-2-yl)-	36.665	0.064	Other compounds	2'-Amino-4'-methoxyacetanilide	82.757	0.0294	Aromatic compounds
Isopropyl phosphine	36.8612	0.0301	Other compounds	Cyclododecene	83.2184	0.209	Hydrocarbons
Pyrazine, 3-ethyl-2,5-dimethyl-	37.1609	2.0661	Pyrazines	Methyl anthranilate	83.5151	2.1134	Esters
Octanoic acid, ethyl ester	37.3431	0.2251	Esters	6-Nitro-o-tolunitrile	84.6024	0.0112	Other compounds
Butanoic acid, 4-butoxy-	37.4209	0.2527	Acids	Propanamide, N-(3-methoxyphenyl)-2,2-dimethyl-	84.665	0.008	Other compounds
1-Heptanol	37.6157	0.114	Alcohols	7-Methyl-5-thiophen-2-yl-1,3-dihydro-thieno[2,3-E]-[1,4]diazepin-2-one	85.2132	0.0307	Other compounds
Pyrazine, 2,6-diethyl-	37.889	0.112	Pyrazines	Sorbic Acid	85.4571	0.1489	Acids
Furfural	38.0053	0.1167	Other compounds	Cyclopentaneacetic acid, 3-oxo-2-pentyl-, methyl ester	85.7837	0.0727	Esters
Pyrazine, tetramethyl-	38.4023	0.045	Pyrazines	Tetracosamethyl-cyclododecasiloxane	85.888	0.1263	Organosilicos compounds
4-Heptanol, 2,6-dimethyl-	38.5891	0.1988	Alcohols	Azelaic dihydrazide	86.484	0.1283	Other compounds

Acetic acid	38.7375	0.261	Acids	Succinic acid, but-3-yn-2-yl 2-methoxy-5-methylphenyl ester	86.672	0.0521	Esters
Pyrazine, 2-ethenyl-6-methyl-	39.0186	0.247	Pyrazines	2,4-Di-tert-butylphenol	87.0107	0.6439	Aromatic compounds
1-Hexanol, 2-ethyl-	39.2334	0.6505	Alcohols	n-Decanoic acid	88.1517	0.469	Acids
Cycloheptasiloxane, tetradecamethyl-	39.5082	0.4309	Organosilicos compounds	Diethyl Phthalate	88.8248	0.0454	Other compounds
Ethanone, 1-(2-furanyl)-	39.813	0.0901	Ketones	4-Pyridinecarbonitrile, 1-oxide	89.4087	0.0401	Heterocyclic compounds
(Z)-Tetradec-11-en-1-yl 2,2,2-trifluoroacetate	40.0622	0.1277	Acids	Hexestrol	90.3676	0.0223	Other compounds
Acridine, 9-methyl-	40.2084	0.2833	Other compounds	Cyclooctasiloxane, hexadecamethyl-	91.1672	0.0207	Organosilicos compounds
Benzaldehyde	40.5983	0.7643	Aldehydes	Homosalate	92.7891	0.0366	Other compounds
2-[(Trimethylsilyl)oxy]-2-{4-[(trimethylsilyl)oxy]phenyl}ethanamine	40.924	0.4142	Other compounds	Quinoline, 4-ethyl-	93.0055	0.0256	Other compounds
Pyrazine, 2-methyl-6-(1-propenyl)-, (E)-	41.2435	0.4803	Pyrazines	Benzamide, 2,4,6-trinitro-N,N-dimethyl-	93.6421	0.003	Amidas
Cyclohexanol, 3,3,5-trimethyl-, cis-	41.4724	0.06	Alcohols	3,5-di-tert-Butyl-4-hydroxybenzaldehyde	93.8697	0.014	Aldehydes
1-Octanol	42.0788	1.2641	Alcohols	4,6-Dichloro-5-cyanopyrimidine	95.2412	0.0692	Heterocyclic compounds
2-Thiaoctane, 4-(9-borabicyclo[3.3.1]non-9-yloxy)-	42.3112	0.0639	Other compounds	Serratinine, 8-acetate	95.6338	0.0174	Other compounds
2,3-Butanediol, [R-(R*,R*)]-	42.5427	1.3455	Other compounds	n-Hexadecanoic acid	95.9707	0.0098	Acids
				3a-(2-Oxocyclohexyl)-dihydrofuro[3,4-c]pyrrole-1,3,4,6-tetrone	96.8341	0.0133	Other compounds

Table S4. Identification of COVs produced by *K. cowanii* Cp1 at 6 h p1 by HS-SPME-GC-MS.

Compounds	Retention Time (min)	Relative Peak Area (%)	Chemical Classes	Compounds	Retention Time (min)	Relative Peak Area (%)	Chemical Classes
**Carbon dioxide**	4.2148	1.5834	Hydrocarbons	Benzaldehyde	40.5945	1.0877	Aldehydes
**Pentane**	4.355	0.3694	Hydrocarbons	4-Trimethylsilyl-9,9-dimethyl-9-silafluorene	40.9261	0.3662	Other compounds
**5-Isoxazolepropanamine, N-methyl-3-(4-nitrophenyl)-**	4.4769	0.4825	Other compounds	Benzaldehyde, 2,4-dimethyl-	41.2367	0.3122	Aldehydes
**n-Hexane**	4.5814	0.2042	Other compounds	Cyclohexanol, 3,3,5-trimethyl-	41.4746	0.0694	Alcohols
**Ethyl ether**	4.6383	0.7497	Ethers	1-Octanol	42.0854	0.8553	Alcohols
**Propane, 2-methoxy-2-methyl-**	4.9493	0.5467	Other compounds	14-Methylpentadec-9-enoic acid methyl ester	42.3522	0.0602	Esters
**Heptane**	5.0886	2.7363	Hydrocarbons	2,3-Butanediol	42.5605	4.1653	Other compounds
**Acetone**	6.3904	1.3664	Ketones	Quinoline, 4-(4-chlorophenoxy)-8-fluoro-2-trifluoromethyl-	42.9027	1.1333	Other compounds
**Cyclotrisiloxane, hexamethyl-**	7.4238	1.241	Other compounds	Phosphonoacetic Acid, 3TMS derivative	43.5433	0.2773	Acids
**Butanal**	7.8062	0.5058	Aldehydes	2-Undecanone	44.2501	0.285	Ketones
**Ethyl Acetate**	8.2457	0.3688	Ácidos carboxílicos	2-Amino-2-oxo-acetic acid, N-[3,4-dimethylphenyl]-, ethyl ester	44.3732	0.0927	Esters
**2-Butanone**	8.5454	1.4304	Ketones	Diethyl carbitol	44.5221	0.2403	Other compounds
**Butanal, 2-methyl-**	9.0651	0.343	Aldehydes	Butyrolactone	44.9055	0.2206	Other compounds
**Butanal, 3-methyl-**	9.2572	1.5338	Aldehydes	Benzeneacetaldehyde	45.7205	0.2205	Aldehydes
**Ethanol, 2-methoxy-, carbonate (2:1)**	9.5915	0.1549	Alcohols	2-Acetylthiazole	45.9046	0.1579	Tioles
**Ethanol**	9.9087	7.5719	Alcohols	Acetophenone	46.055	0.2117	Ketones
**2-Butanone, 3-methyl-**	12.0638	0.0199	Ketones	2-Furanmethanol	46.3652	0.578	Alcohols
**2,3-Butanedione**	12.5144	2.0864	Ketones	Pyrazine, 2,5-dimethyl-3-(3-methylbutyl)-	46.746	0.6693	Pyrazines
**Trichloroethylene**	13.0492	0.5284	Other compounds	Benzoic acid, 2-formyl-	47.097	0.2009	Acids
**Methyl isocyanide**	13.5895	0.0562	Other compounds	Benzaldehyde, 2-hydroxy-	47.7644	0.1597	Aldehydes
**Cyclotetrasiloxane, octamethyl-**	14.2547	1.0141	Other compounds	.alpha.-D-Galactopyranoside, methyl	49.0932	0.4892	Other compounds
**Trichloromethane**	14.7774	0.2041	Other compounds	N-Isobutyl-11-(3,4-methylenedioxyphenyl)-2E,4E,10E-undecatrienoic amide	49.5524	0.0899	Other compounds
**2-Oxo-4-phenyl-6-(4-chlorophenyl)-1,2-dihydropyrimidine**	14.9662	0.1407	Other compounds	5-[2-(Dimethoxyphosphonyl)acetoxy]hex-2-enoic acid	50.0029	0.131	Acids
**Toluene**	15.4417	0.245	Hydrocarbons	Acetamide	50.7633	0.0948	Other compounds
**Butanoic acid, ethyl ester**	15.694	0.1706	Esters	1,4-Benzenediol, 2,5-dimethyl-	51.6031	0.059	Alcohols
**Disulfide, dimethyl**	17.1472	0.6794	Other compounds	Cystine	51.8276	0.0424	Other compounds
**Acetic acid, butyl ester**	17.751	0.1166	Esters	1-Decanol	52.5799	1.7302	Alcohols
**Hexanal**	18.0959	0.1403	Aldehydes	Methyl salicylate	53.5142	0.0372	Esters
**(S)-(-)-1,2,4-Butanetriol, 4-trifluoroacetate**	18.3342	0.151	Alcohols	9-Decen-1-ol	56.413	0.2841	Alcohols
**2-Butenal, 2-methyl-**	18.6643	0.1576	Other compounds	2-Hexanone, 4-methyl-	56.8369	0.0577	Ketones
**1-Propanol, 2-methyl-**	19.3076	0.0609	Alcohols	1H-Indenol	57.1242	0.0466	Other compounds
**Ethylbenzene**	20.3083	0.059	Other compounds	9-Decen-1-yl acetate	58.0169	0.048	Esters
**3-Methylheptyl acetate**	20.7453	0.2274	Other compounds	Cyclononasiloxane, octadecamethyl-	58.1858	0.1251	Other compounds
**Diethyl phosphite**	21.0175	0.025	Other compounds	1-Naphthalenol	59.141	0.0381	Alcohols
**o-Xylene**	21.2181	0.1476	Hydrocarbons	Benzyl alcohol	60.9297	0.5091	Alcohols

**Pentanoic acid, ethyl ester**	21.4566	0.2803	Esters	Butanoic acid, butyl ester	61.6643	0.1711	Esters
**1-Butanol**	21.9567	1.6217	Alcohols	Propanoic acid, 2-methyl-, hexyl ester	62.9285	0.0838	Esters
**Pyridine**	23.5129	0.0235	Other compounds	.alpha.-d-Ribopyranoside, methyl	63.4589	0.3882	Other compounds
**Cyclopentasiloxane, decamethyl-**	23.866	0.79	Other compounds	Acetic acid, dichloro-, methyl ester	64.0105	0.1088	Esters
**p-Xylene**	24.3024	0.1304	Hydrocarbons	Phenylethyl Alcohol	64.413	0.1258	Alcohols
**D-Limonene**	25.1621	0.5267	Other compounds	2,2-Dimethyl-3-heptanone	67.3475	0.068	Ketones
**1-Butanol, 3-methyl-**	25.4166	4.9428	Alcohols	Ethyl 2-chloro-3,3,3-trifluoro-2-isovaleramidopropionate	69.0587	0.073	Other compounds
**Thiazole**	27.3043	0.076	Tioles	Ethanone, 1-(1H-pyrrol-2-yl)-	69.3391	0.0246	Ketones
**3-Buten-1-ol, 3-methyl-**	27.5937	0.047	Alcohols	Cyclododecane	70.5476	2.7441	Hydrocarbons
**1,3,5,7-Cyclooctatetraene**	27.9557	0.0623	Other compounds	Acetamide, 2-(4-methoxyphenyl)-N-isobutyl-	71.2623	0.0273	Other compounds
**Pyrazine, methyl-**	28.2139	1.3549	Pyrazines	2-Pyrrolidinone	71.8334	0.0351	Other compounds
**Benzene, 4-ethyl-1,2-dimethyl-**	29.1222	0.1246	Other compounds	Phenol	72.0354	0.0348	Aromatic compounds
**N-{3-Isopropyl-[1,2,4]triazolo[3,4-b][1,3,4]thiadiazol-6-yl}benzamide**	29.2902	0.064	Other compounds	Phenol, O-(ethylsulfinyl)-	72.3273	0.0861	Aromatic compounds
**Acetoin**	29.6282	6.0438	Ketones	Heptanoic acid	72.8902	0.1672	Acids
**Octanal**	30.0686	0.0886	Aldehydes	Cyclodecasiloxane, eicosamethyl-	73.1743	0.1781	Other compounds
**2-Propanone, 1-hydroxy-**	30.3109	0.3178	Hydrocarbons	10-Undecen-1-ol	73.9515	0.1347	Alcohols
**Pyrazine, 2,5-dimethyl-**	31.1047	9.1536	Pyrazines	4,6-Dichloro-5-cyanopyrimidine	76.152	12.2595	Other compounds
**Pyrazine, 2,6-dimethyl-**	31.4365	0.4572	Pyrazines	1,1'-Biphenyl, 3-methyl-	76.9681	0.0315	Other compounds
**Pyrazine, ethyl-**	31.7829	0.1468	Pyrazines	2,6-Bis(1,1-dimethylethyl)-4-(1-oxopropyl)phenol	78.1584	0.0972	Other compounds
**Cyclohexasiloxane, dodecamethyl-**	32.2415	0.3424	Other compounds	3-(tert-Butyl)-4-methoxyphenyl acetate	78.2989	0.0506	Other compounds
**Methyl 2,5,8,11-tetraoxatridecan-13-oate**	32.4655	0.027	Other compounds	Octanoic acid	78.5868	1.2376	Acids
**1-Hexanol**	32.8437	0.146	Alcohols	Benzenamine, 2,5-dimethyl-	79.7828	0.0874	Other compounds
**Fumaric Acid**	33.2526	0.0469	Acids	Cyclooctasiloxane, hexadecamethyl-	80.5348	0.0498	Other compounds
**Dimethyl trisulfide**	33.9942	0.0942	Other compounds	1H-Pyrazole, 3-methyl-	81.346	0.0324	Other compounds
**Undecanoic acid, 2,4,6-trimethyl-, methyl ester**	34.3096	0.1454	Esters	7-Hexadecene, (Z)-	81.8691	0.0937	Other compounds
**Pyrazine, 2-ethyl-6-methyl-**	34.6082	0.5342	Pyrazines	Cyclononasiloxane, octadecamethyl-	82.2844	0.0924	Other compounds
**2-Nonanone**	34.9646	0.2318	Ketones	4-Butoxycarbonyl-4'-formylaminoazobenzene	82.4921	0.1365	Other compounds
**Pyrazine, trimethyl-**	35.1289	0.8557	Pyrazines	2,5-Bis(5-tert-butyl-2-benzoxazolyl)thiophene	82.725	0.0567	Other compounds
**Pyrazine, 2-methyl-5-(1-methylethyl)-**	35.676	0.1808	Pyrazines	1,7-Hexadecadiene	83.0452	0.2306	Other compounds
**Isonicotinamide, N,N-didecyl-**	36.6483	0.0586	Other compounds	Nonanoic acid	83.4135	2.3664	Acids
**Pyrazine, 3-ethyl-2,5-dimethyl-**	37.1587	1.1049	Pyrazines	Oxazepam, 2TMS derivative	85.8652	0.103	Other compounds
**Methional**	37.5356	0.0646	Other compounds	Ethanone, 1-(4,5-dihydro-2-thiazolyl)-	86.5769	0.1361	Ketones
**Formic acid, heptyl ester**	37.6136	0.0678	Esters	2,4-Di-tert-butylphenol	87.0063	0.1254	Aromatic compounds
**Acetic acid**	37.9195	0.4931	Acids	1(2H)-Naphthalenone, 3,4-dihydro-2-(phenylmethylene)-	87.447	0.0976	Other compounds
**3-Furaldehyde**	37.9922	2.0136	Aldehydes	n-Decanoic acid	88.1729	0.6736	Acids
**4-Heptanol, 2,6-dimethyl-**	38.5802	0.7236	Alcohols	Diethyl Phthalate	88.8125	0.0642	Other compounds
**1-Hexanol, 2-ethyl-**	39.2427	0.531	Alcohols	2-Oxoadamantane-1-carboxamide, N-(1-phenylethyl)-	89.3863	0.0659	Other compounds
**Cycloheptasiloxane, tetradecamethyl-**	39.496	0.3015	Other compounds	Benzenesulfonamide, N-(adamantan-1-yl)methyl-4-tert-butyl-	90.3503	0.019	Other compounds

**Ethanone, 1-(2-furanyl)-**	39.8133	0.085	Ketones	Tetracosamethyl-cyclododecasiloxane	91.1365	0.0452	Other compounds
**1H-Indene, octahydro-, cis-**	40.0534	0.092	Other compounds	tert-Butyldimethylsilyl 3-chloro-5-methoxy-4-(2,2,2-trifluoroacetoxy)benzoate	95.4524	0.041	Other compounds
**2-Mercapto-4-phenylthiazole**	40.2064	0.3456	Tioles	Dodecanoic acid	96.0639	0.0348	Acids
				2-Propanone, 1,1,3-trichloro-	97.6867	0.0196	Ketones

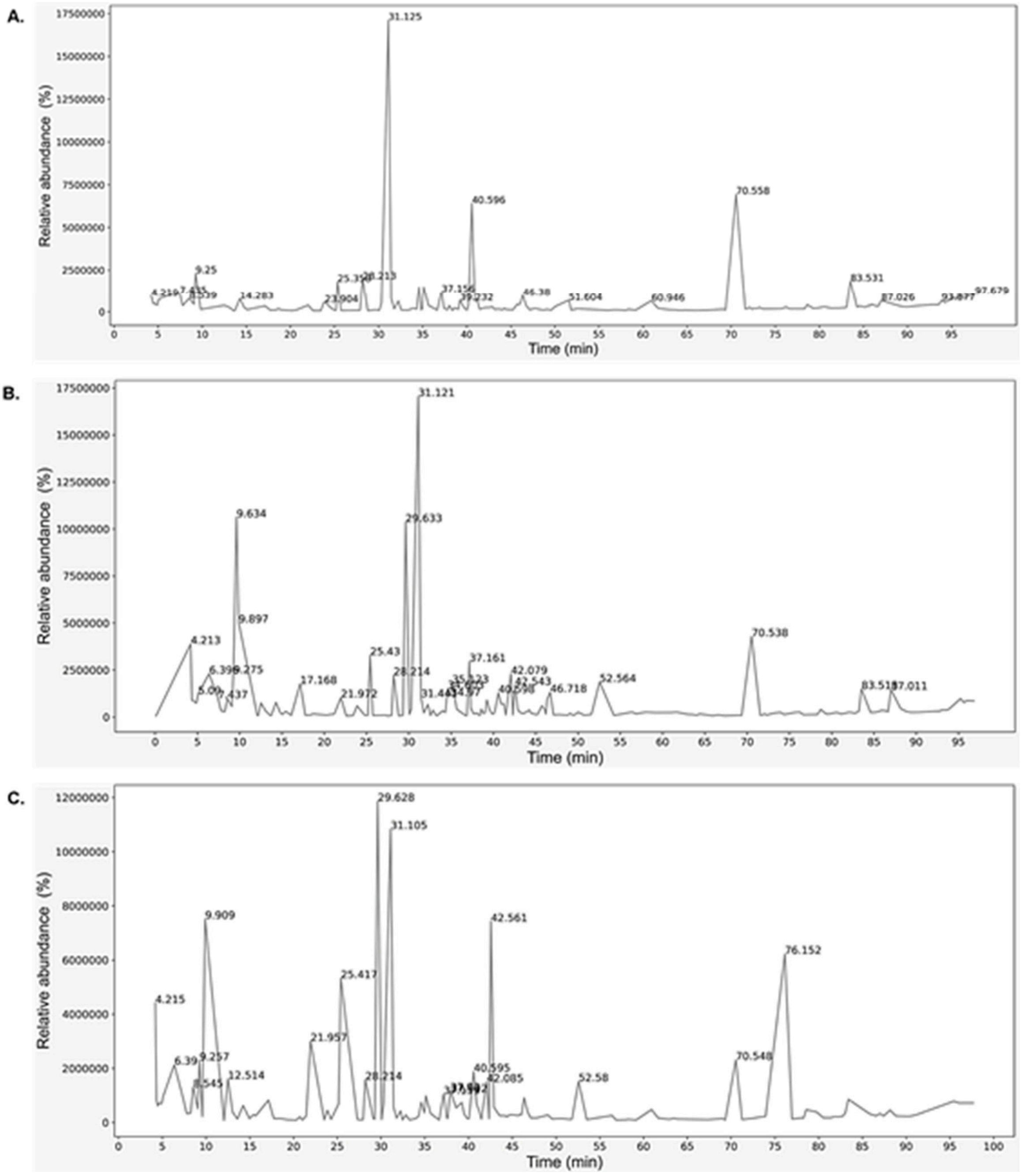

Figure S5. Detection of VOCs produced by *K. cowanii* Cp1. The main volatile organic compounds emitted by the *K. cowanii* Cp1 strain were identified during bacterial growth phases: 2 hours, 4 hours and 6 hours. Among the main VOCs that were detected at 2 h were pyrazine, 2,5-dimethyl-, cyclododecane, benzaldehyde, methyl anthranilate, pyrazine, methyl-, pyrazine, Butanal, 3-methyl-, pyrazine, 2-ethyl-5-methyl-, pyrazine, 3-ethyl-2,5-dimethyl- and 1-Hexanol, 2-ethyl-. **B.** The VOCs identified at 4 h were pyrazine, 2,5-dimethyl-, isopropyl alcohol, acetoin, cyclododecane, 1-Butanol, 3-methyl-, pyrazine, 3-ethyl-2,5-dimethyl-, 1-Octanol, pyrazine, methyl-, disulfide, dimethyl and 1-Butanol. **C.** Finally, the compounds that were produced at 6 h included acetoin, pyrazine, 2,5-dimethyl-, 2,3-Butanediol, ethanol, 4,6-Dichloro-5-cyanopyrimidine, 1-Butanol, 3-methyl-, 1-Butanol, cyclododecane, pyrazine, methyl- and benzaldehyde.
